# Mechanisms for self‐templating design of micro/nanostructures toward efficient energy storage

**DOI:** 10.1002/EXP.20210237

**Published:** 2022-05-31

**Authors:** Zengyu Hui, Jianing An, Jinyuan Zhou, Wei Huang, Gengzhi Sun

**Affiliations:** ^1^ Institute of Flexible Electronics (IFE) Northwestern Polytechnical University (NPU) Xi'an P. R. China; ^2^ Institute of Advanced Materials (IAM) Nanjing Tech University (NanjingTech) Nanjing P. R. China; ^3^ Institute of Photonics Technology Jinan University Guangzhou P. R. China; ^4^ School of Physical Science and Technology Lanzhou University Lanzhou P. R. China

**Keywords:** electrode materials, energy storage, micro/nanostructures, self‐templating

## Abstract

The ever‐growing demand in modern power systems calls for the innovation in electrochemical energy storage devices so as to achieve both supercapacitor‐like high power density and battery‐like high energy density. Rational design of the micro/nanostructures of energy storage materials offers a pathway to finely tailor their electrochemical properties thereby enabling significant improvements in device performances and enormous strategies have been developed for synthesizing hierarchically structured active materials. Among all strategies, the direct conversion of precursor templates into target micro/nanostructures through physical and/or chemical processes is facile, controllable, and scalable. Yet the mechanistic understanding of the self‐templating method is lacking and the synthetic versatility for constructing complex architectures is inadequately demonstrated. This review starts with the introduction of five main self‐templating synthetic mechanisms and the corresponding constructed hierarchical micro/nanostructures. Subsequently, the structural merits provided by the well‐defined architectures for energy storage are elaborately discussed. At last, a summary of current challenges and future development of the self‐templating method for synthesizing high‐performance electrode materials is also presented.

## Graphical abstract

The micro/nanostructure design via self‐templating method offers a viable way to significantly improve the electrochemical performances of functional materials. This review introduces five main self‐templating mechanisms and compares the merits of different micro/nanostructures for energy storage. Furthermore, a summary of current challenges and the prospect of self‐templating strategy for constructing high‐performance electrode materials are also presented.

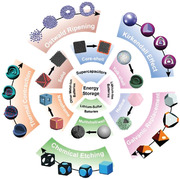

## 1 INTRODUCTION

The sharp depletion of fossil fuels and the drastic energy consumption increase have driven the pursuit of renewable and green energy resources and the development of electrochemical energy storage (EES) technologies.^[^ ^1^, ^2^, ^3^, ^4^, ^5^, ^6^, ^7^ ^]^ The mainstream of current EES devices lies in batteries and supercapacitors that have complementary charge storage properties owing to their fundamentally different mechanisms. Generally, batteries exhibit high energy densities which can be attributed to the large amount of stored charges through diffusion‐limited redox reactions.^[^ ^8^, ^9^, ^10^, ^11^, ^12^, ^13^ ^]^ On the contrary, benefiting from the electric double‐layer capacitive behavior or the rapid surface/subsurface redox reactions, supercapacitors can deliver high power densities.^[^ ^14^, ^15^, ^16^, ^17^, ^18^ ^]^ Therefore, developing novel electrode materials that are capable of simultaneously achieving high charge storage capacity and rapid charge/discharge ability is valid to enable high‐performance and cost‐effective EES devices.

**FIGURE 1:**
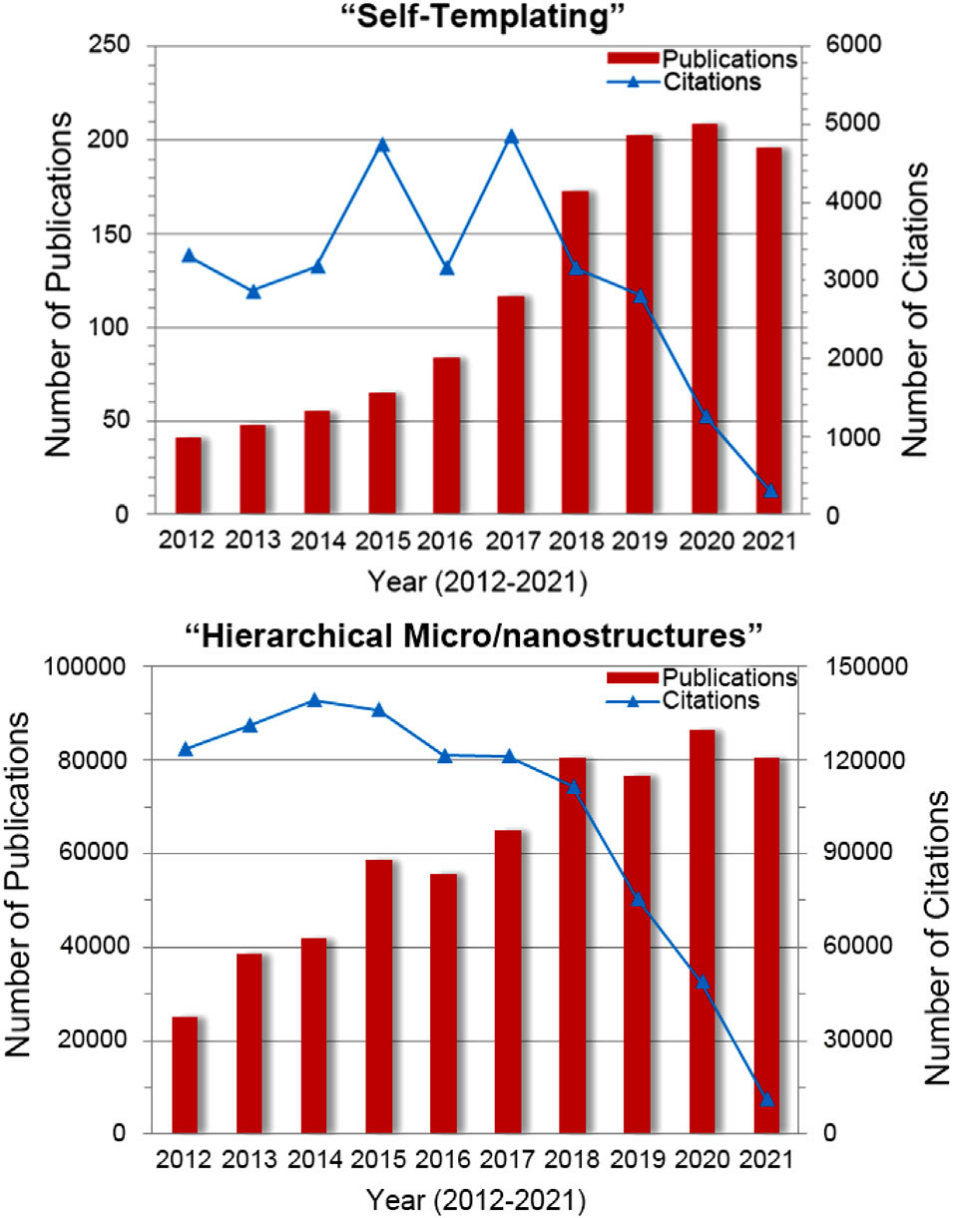
Plots of the publication and citation numbers on “self‐templating” and “hierarchical micro/nanostructures” per year in the past decade

As plotted in Figure 1, an increasing number of researches show that the structural design of electrode materials provides an efficient route to achieve these goals.^[^ ^19^, ^20^, ^21^ ^]^ Typically, the creation of hierarchical hollow architectures offers electrode materials the structural merits of high porosity, low density, and large surface area.^[^ ^22^ ^]^ Specifically, the nanoscale subunits can shorten the diffusion path for fast charge and discharge; the hollow shells can minimize the aggregation of the embedded nanoparticles and alleviate the huge volume expansion/shrinkage during cycling; the interior nanostructures can enhance the overall volumetric energy by increasing the weight percentage of active components.^[^ ^23^ ^]^ In another aspect, solid structures with textured surfaces possess stable physical and chemical properties, enlarged accessible area, and tunable porosity, providing rich ion storage interfaces and enhanced ion transport.^[^ ^24^ ^]^

Enormous attentions have been paid for controllably synthesizing active materials with hierarchical micro/nanostructures.^[^ ^25^, ^26^, ^27^, ^28^, ^29^, ^30^, ^31^, ^32^, ^33^ ^]^ Template‐assisted approaches, for example, hard‐,^[^ ^34^, ^35^, ^36^, ^37^, ^38^ ^]^ soft‐,^[^ ^39^, ^40^, ^41^, ^42^, ^43^ ^]^ and self‐templating methods^[^ ^44^, ^45^, ^46^ ^]^ are the widely adopted preparation approaches. Hard‐templating is a straightforward strategy, which normally employs precursor entities with well‐defined geometry as the hard templates.^[^ ^47^ ^]^ Target hierarchical micro/nanostructures can be obtained by selectively eliminating the sacrificial templates using chemical etching or calcination at a high temperature.^[^ ^48^ ^]^ However, the produced structures are susceptible to damage during the harsh template removal steps. Soft materials including emulsion droplets,^[^ ^49^, ^50^, ^51^ ^]^ vesicles/micelles,^[^ ^52^, ^53^, ^54^ ^]^ and gas bubbles^[^ ^55^, ^56^, ^57^ ^]^ have been used as the alternative templates. Although the soft‐templating preparation process is mild because of the avoidance of template removal, the uniformity control of the products is challenging due to the instability of the soft templates. Comparably, self‐templating is more facile and flexible because the final structures are directly derived from the source template materials through chemical reactions.^[^ ^58^, ^59^, ^60^, ^61^, ^62^ ^]^ The self‐confined strategy not only simplifies the synthesis procedure with reduced cost, but also enables good control over the uniformity of the products.^[^ ^44^, ^63^ ^]^ During the past decade, the field of preparing well‐defined architectures using self‐templating approach has drawn great attention, together with the extensive researches on the corresponding structural versatility and superiority for energy storage applications. In this article, we begin with the comprehensive introduction of the general self‐templating synthetic routes according to the formation mechanisms, particularly focusing on (i) Ostwald ripening, (ii) Kirkendall effect, (iii) galvanic replacement, (iv) chemical etching, and (v) template contraction and transformation. Next, the classified hierarchical micro/nanostructures, including surface‐textured solid, core–shell, yolk–shell, single‐shelled hollow and multi‐shelled, and nanoframe configurations, are presented in sequence; their applications in EES devices for enhancing the performances are thoroughly addressed. At last, the summary of current challenges and our perspectives on preparing hierarchical micro/nanostructures via self‐templating principles toward efficient energy storage are provided.

## 2 SELF‐TEMPLATING SYNTHETIC MECHANISMS

Self‐templating method provides a direct synthetic route free from additional templates, because the precursor materials function both as the source and the template for regulating the composition and structures of the final products. The method has demonstrated efficiency and diversity in constructing various hierarchical micro/nanostructures with complex compositions. The generally adopted self‐templating mechanisms encompass Ostwald ripening, Kirkendall effect, galvanic replacement, chemical etching, and template contraction and transformation. In this section, we will elaborately introduce each synthetic method and compare their advantages/disadvantages for preparing hierarchical micro/nanostructures (as listed in Table 1).

**TABLE 1:** The comparison between five main self‐templating synthetic mechanisms

Merits				
Mechanisms	Material size	Material crystallinity	Reaction condition	Application diversity
Ostwald ripening	Large	Low	Harsh	Wide
Kirkendall effect	All‐Scale	High	Harsh	Narrow
Galvanic replacement	All‐Scale	Low	Mild	Narrow
Chemical etching	All‐Scale	Low	Harsh	Wide
Template contraction and transformation	All‐Scale	Low	Harsh	Wide

### 2.1 Ostwald ripening

**FIGURE 2:**
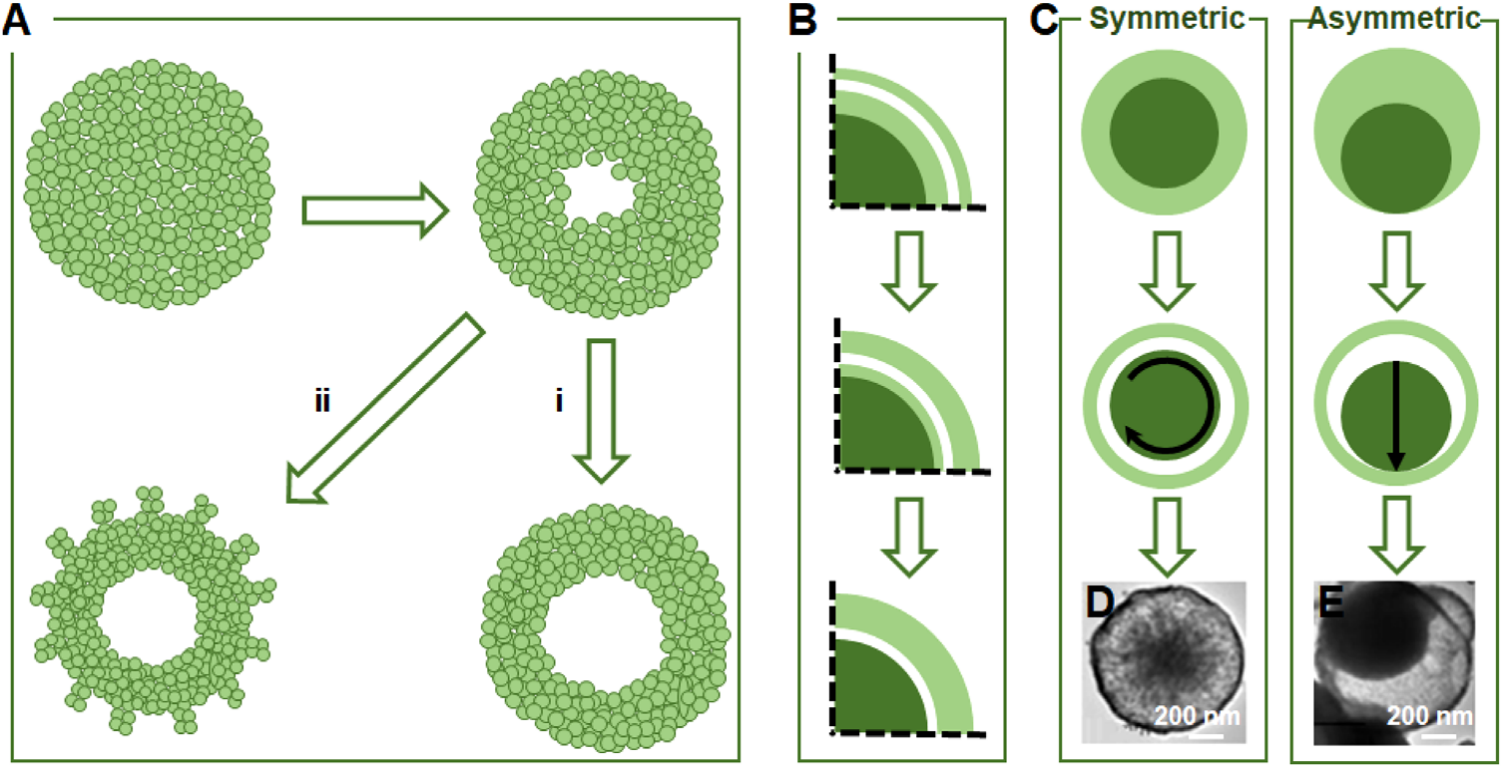
(A) The Ostwald ripening‐induced generation of two types of hollow configurations. (B) Schematic formation of the void spaces between core and shell. Reproduced with permission.^[^ ^64^ ^]^ Copyright 2004, American Chemical Society. (C) Schemes of symmetric and asymmetric Ostwald ripening mechanisms to produce homogeneous and semi‐hollow core–shell architectures, respectively. TEM images of (D) a ZnS core–shell nanosphere and (E) a Co_3_O_4_ semi‐hollow core–shell nanosphere. Reproduced with permission.^[^ ^65^ ^]^ Copyright 2005, Wiley‐VCH

Ostwald ripening describes a process by which small crystallites are dissolved and re‐deposited on the surface of large species. It has been proven as an effective way to obtain diverse hollow structures, and was first adopted by Yang et al. to prepare TiO_2_ hollow nanospheres with the hydrolysis of TiF_4_ under a long‐time hydrothermal treatment.^[^ ^64^ ^]^ As illustrated in Figure 2A, the precursor was initially transformed into solid‐state TiO_2_ nanospheres composed of small crystallites. The core crystallites were dissolved and re‐deposited on the outer shell surface as a function of the reaction time, following the Ostwald ripening mechanism. Two types of hollow configurations were observed, which were respectively classified as type (i) having a dense and smooth surface, and type (ii) having extrudes on the less compact surface, which was caused by a “refilling” process.

The same research group further investigated the construction of complex hollow structures by virtue of the symmetric and asymmetric Ostwald ripening mechanisms, which depend on the chemico‐physical properties of different components. As demonstrated in Figure 2B, the starting solid aggregate comprised a large and closely packed central region (dark areas in Figure 2B), and a small and loosely packed surface region (light areas in Figure 2B). Once the chemical reaction was triggered, the crystallites on the outermost surface served as the nucleation seeds for solid evacuation and subsequent recrystallization. As the hydrothermal treatment continued, the outer shells grew larger while the core region trimmed down, creating void spaces in between. If the solid evacuation occurred over the whole shell surface by following a symmetric Ostwald ripening mechanism, a rotatable core was detached from the shell, forming a homogeneous core–shell structure (left scheme in Figure 2C). This was experimentally evidenced by the observation of ZnS core–shell nanospheres in the TEM image (Figure 2D). On the contrary, if the solid evacuation only took place in a certain region by proceeding in an asymmetric manner, a tumbler‐type mass imbalance was induced, producing a basketlike core–shell structure (right scheme in Figure 2C). This was experimentally validated by the synthesis of Co_3_O_4_ semi‐hollow core–shell nanospheres (TEM image in Figure 2E). Hence, the Ostwald ripening process is tailorable for designing hollow patterns of the resultant structures.^[^ ^65^ ^]^

### 2.2 Kirkendall effect

**FIGURE 3:**
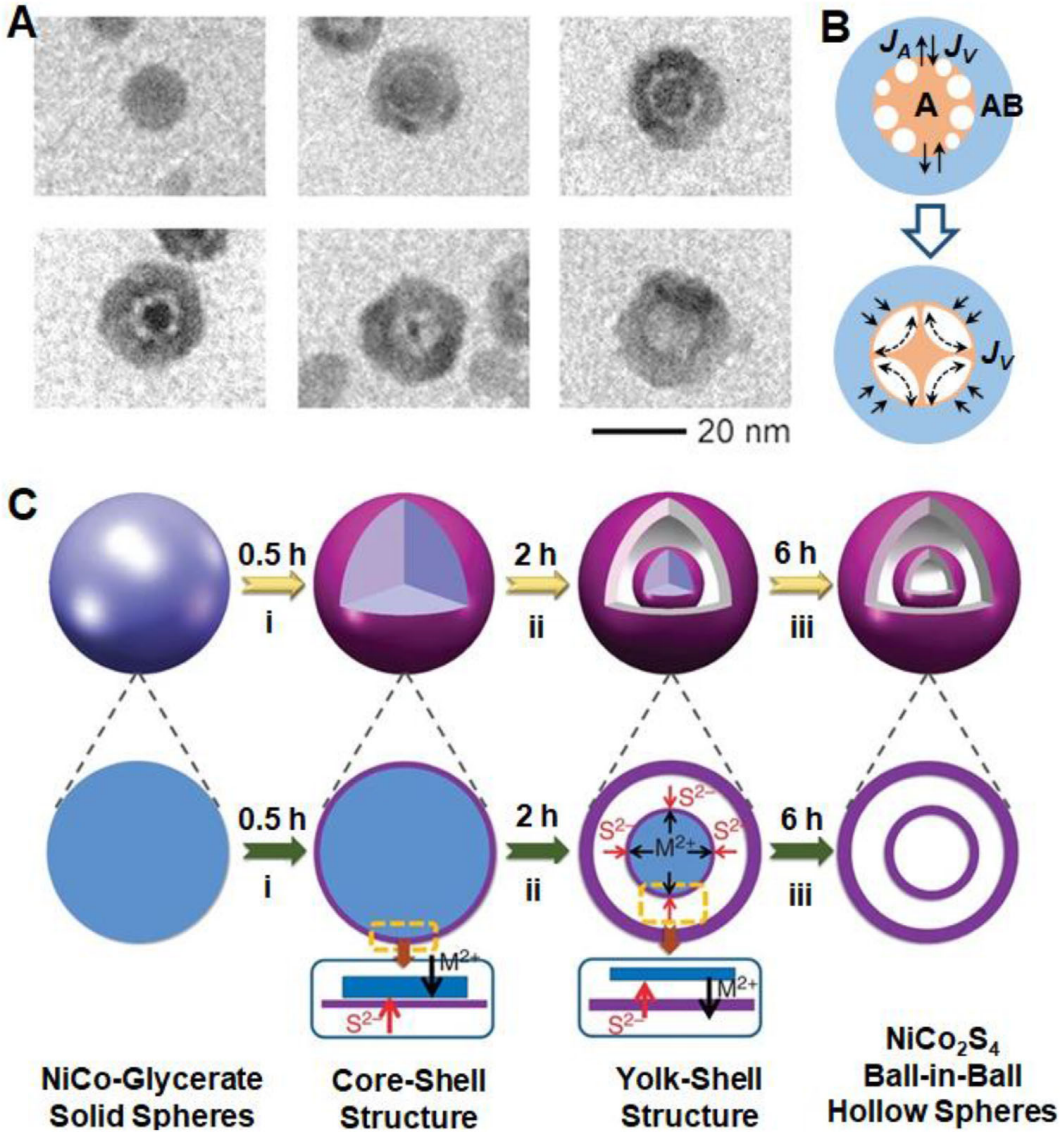
(A) The structural evolution of CoSe hollow nanocrystals. Reproduced with permission.^[^ ^66^ ^]^ Copyright 2004, American Association for the Advancement of Science. (B) A well‐established model for the generation of hollow structures according to the “Kirkendall effect” and “surface diffusion effect.” Reproduced with permission.^[^ ^67^ ^]^ Copyright 2007, American Chemical Society. (C) The evolution of NiCo_2_S_4_ hollow spheres with a “ball‐in‐ball” configuration. Reproduced with permission.^[^ ^70^ ^]^ Copyright 2015, Royal Society of Chemistry

In metallurgy, a classical phenomenon describing the motion of the interfacial atoms between two materials resulting from their different diffusion rates is known as the Kirkendall effect. In nanoscale domain, the Kirkendall effect also finds applications for constructing yolk–shell structures, hollow structures, and nanoframes in self‐templating routes. Early in 2004, Yin et al. first synthesized hollow nanocrystals of Co‐based compounds by taking advantage of the Kirkendall effect. For example, CoSe hollow nanocrystals were synthesized by simply mixing a suspension of Co and Se in o‐dichlorobenzene at high temperatures.^[^ ^66^ ^]^ The structural evolution of CoSe hollow nanocrystals is illustrated in Figure 3A. The outward diffusion of Co atoms to the shell induced the generation and growth of lattice vacancies (Figure 3B),^[^ ^67^ ^]^ ultimately forming hollow structures. In addition, CoS hollow nanospheres, CoO hollow nanospheres, and Pt@CoO yolk–shell nanostructures were also synthesized using the same method.

The Kirkendall effect properly explained the diffusion and exchange reactions between metal cations and sulfide/oxygen anions during the sulfidation/oxidation processes.^[^ ^68^, ^69^ ^]^ Shen et al. synthesized NiCo_2_S_4_ hollow nanoparticles with a “ball‐in‐ball” configuration by using the anion‐exchange method.^[^ ^70^ ^]^ As schematically illustrated in Figure 3C, NiCo‐glycerate solid nanoparticles were employed as the precursor to react with the S^2−^ ions, forming a NiCo‐glycerate@NiCo_2_S_4_ core–shell structure in the initial stage. The growth of outer NiCo_2_S_4_ shell continued as the sulfidation proceeded, eventually terminated when the diffusion of metal cations was prohibited by forming a void space. Consequently, a secondary shell was generated until the total consumption of the NiCo‐glycerate, thus producing a NiCo_2_S_4_ hollow nanosphere with a unique “ball‐in‐ball” configuration.

### 2.3 Galvanic replacement

**FIGURE 4:**
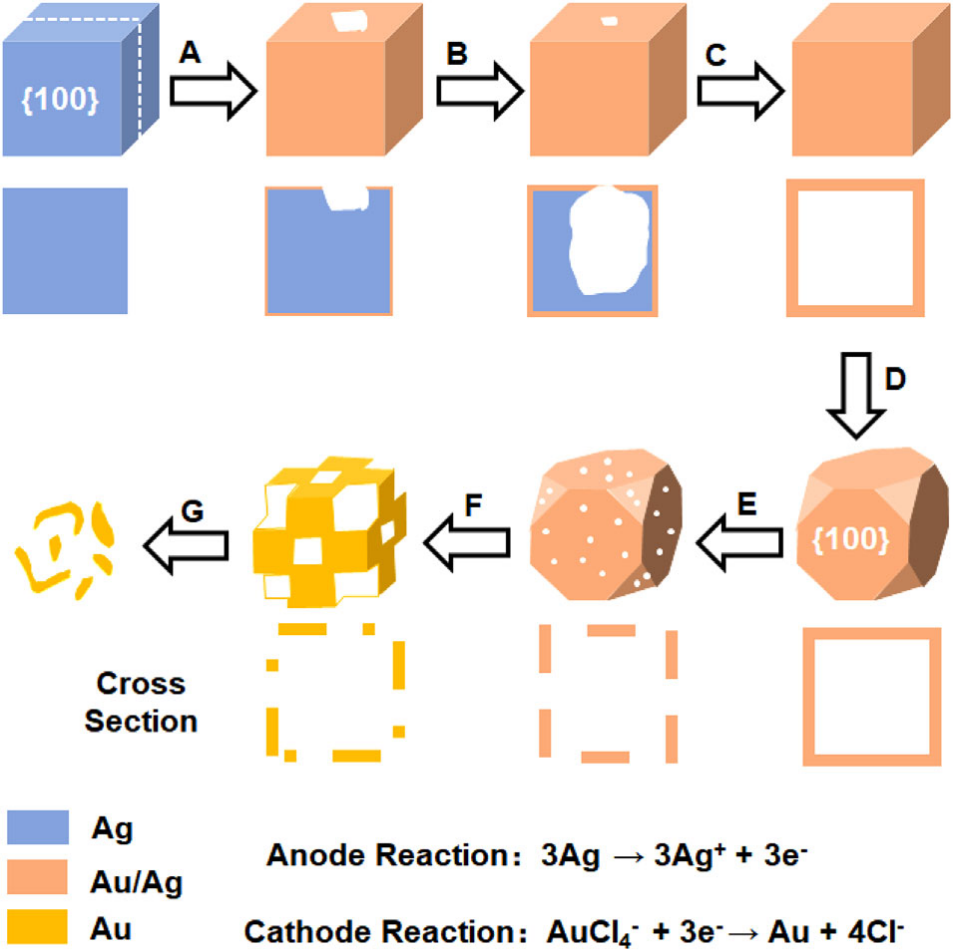
The reaction between HAuCl_4_ aqueous solution and Ag nanocube via galvanic replacement. Reproduced with permission.^[^ ^84^ ^]^ Copyright 2004, American Chemical Society

Galvanic replacement can be simply understood as the reaction occurred between one metal, which functions as the sacrificial template, and other metal ions in solution that have higher electrochemical potentials.^[^ ^71^ ^]^ The potential difference drives the reduction and deposition of metal ions while simultaneously enabling the oxidative dissolution of the sacrificial templates.^[^ ^72^, ^73^ ^]^ Therefore, by adjusting the type and amount of the salt precursors, the composition of the resultant products can be facilely tailored, while their morphology and internal structures can be finely engineered by the sacrificial template with pre‐designed nanostructures.^[^ ^74^ ^]^ Up to now, the galvanic replacement strategy has demonstrated the versatility to generate advanced multifunctional nanostructures based on a large variety of metal nanocrystals (such as Ag,^[^ ^75^, ^76^ ^]^ Co,^[^ ^77^, ^78^ ^]^ Cu,^[^ ^79^, ^80^ ^]^ etc.) with relatively low electrode potential and alterable colloid chemistry. Xia et al. investigated the synthesis of diverse Au hollow nanostructures through galvanic replacement between HAuCl_4_ and Ag in their serial works.^[^ ^81^, ^82^, ^83^ ^]^ A typical reaction process with Ag nanocubes as the starting template material is exemplified in Figure 4.^[^ ^84^ ^]^ Upon contacting the HAuCl_4_ aqueous solution, active sites such as the defects on Ag nanocube surface started to dissolve by oxidation, generating a hole locally. At the same time, the Au ions were reduced, forming a Au shell. With the Ag dissolution and Au coverage proceeding, hollow nanostructures were formed. Since the interdiffusion rates of Ag and Au atoms were almost the same, homogeneous Ag–Au alloys were thermodynamically favorable, leading to a Ag–Au alloy shell. By further adding HAuCl_4_ to the reaction system, Ag in the alloy was selectively etched by following a dealloying process, resulting in a morphological reconstruction to release the surface energy. Following these pioneering works, various hollow nanostructures including nanospheres, nanopolyhedrons, nanocolumns, nanotubes, and nanorattles were synthesized by means of the galvanic replacement reactions.

### 2.4 Chemical etching

Chemical etching is a subtractive manufacturing process that can selectively remove certain components to construct target structures via chemical reactions. According to the chemical reaction types, chemical etching can be categorized as redox etching and non‐redox etching. It is widely used to construct various configurations including surface‐textured solid structures, core–shell structures, yolk–shell structures, single‐shelled hollow structures, multi‐shelled structures, and nanoframes.

**FIGURE 5:**
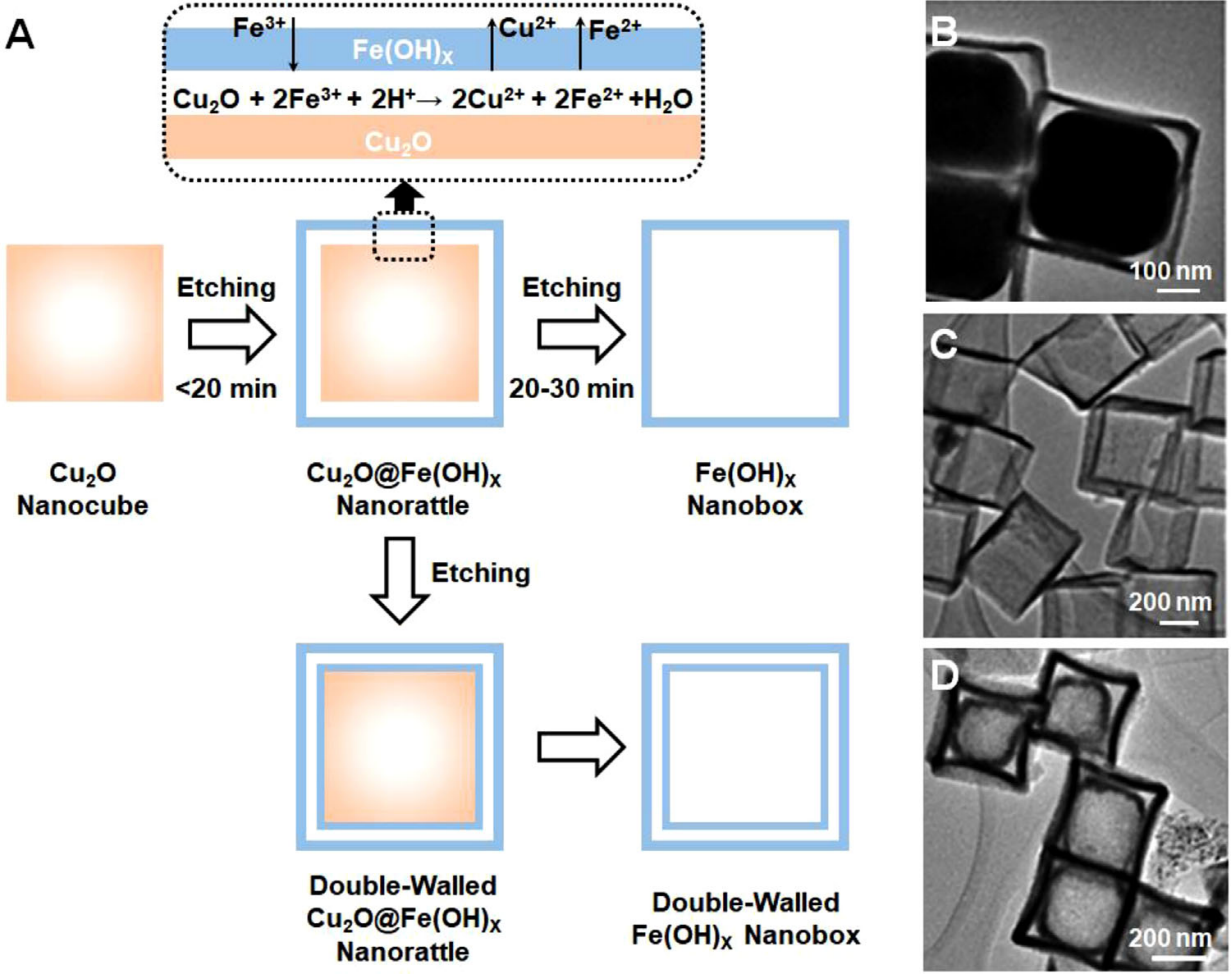
(A) Schematic diagram showing the transformation from Cu_2_O nanocubes into hollow nanoboxes with single‐wall or double‐wall configurations via redox etching. TEM images of (B) Cu_2_O@Fe(OH)x nanorattles, (C) Fe(OH)x nanoboxes, and (D) double‐walled Fe(OH)x nanoboxes. Reproduced with permission.^[^ ^85^ ^]^ Copyright 2010, American Chemical Society

Redox etching is normally employed to obtain nanostructures by reactions between redox couples. For example, Fe(OH)*_x_* hollow structures were prepared by chemical etching of Cu_2_O templates and subsequent hydrolysis reactions.^[^ ^85^ ^]^ As illustrated in Figure 5A, Cu_2_O nanocrystals can be readily oxidized by Fe^3+^ ions having a higher potential, generating unstable Fe^2+^ ions, which were then transformed into Fe(OH)*_x_* precipitates upon hydrolysis in an aqueous solution. The Cu_2_O core was consumed as the reaction proceeded, leaving a Fe(OH)*_x_* shell duplicating the morphology of the sacrificial template. Consequently, by pre‐designing the dimension of the templates, it is feasible to well control the topology of the resultant products. In addition, the interiors were facilely engineered by multiple redox etching of the remaining cores (Figure 5B), forming complex architectures such as Fe(OH)x nanoboxes (Figure 5C), and box‐in‐box nanostructures (Figure 5D).

**FIGURE 6:**
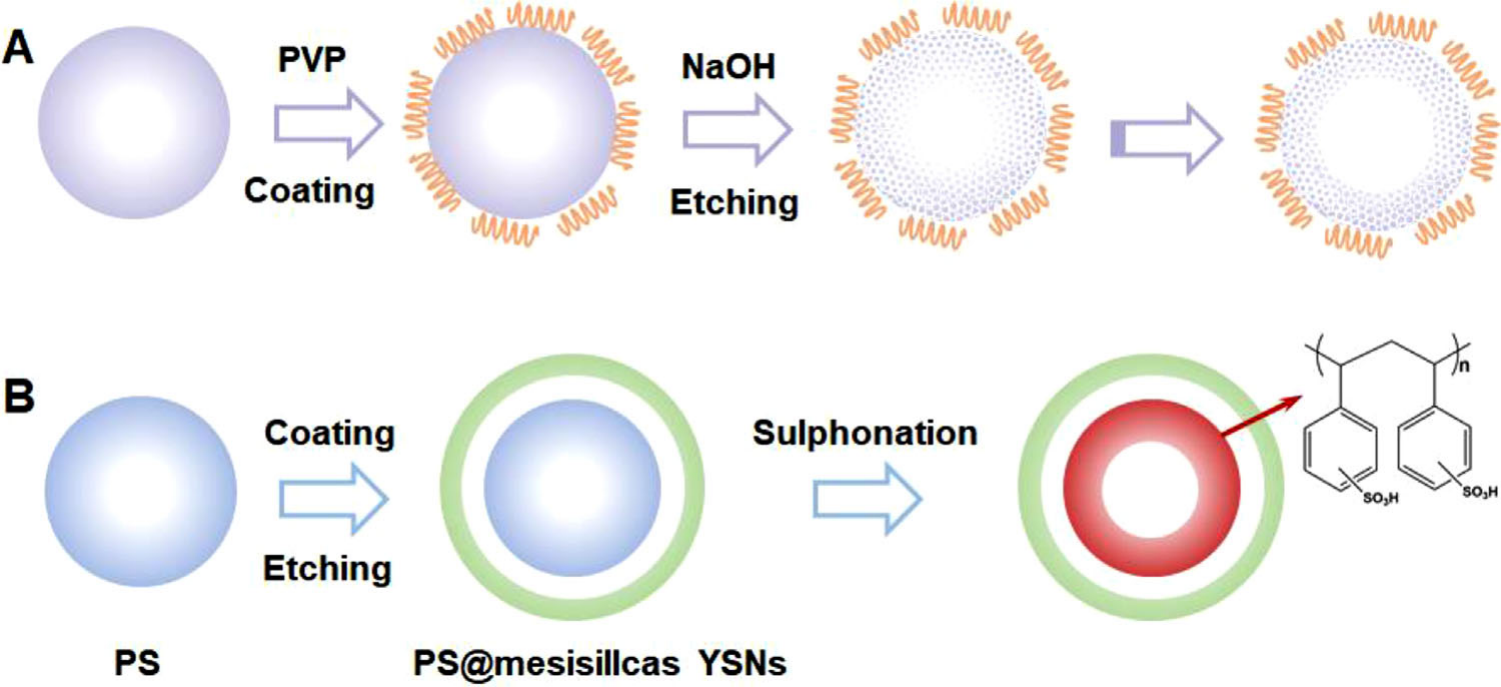
Conceptual demonstrations of (A) “surface‐protected etching” and (B) “organosilane‐assisted etching” for preparing hollow nanostructures with permeable shells from solid nanospheres, respectively. Reproduced with permission.^[^ ^86^ ^]^ Copyright 2008, American Chemical Society. Reproduced with permission.^[^ ^87^ ^]^ Copyright 2014, American Chemical Society

Non‐redox etching refers to the construction of complex structures via non‐redox reactions. A typical demonstration was exemplified by Yin and co‐workers where a poly(vinyl pyrrolidone) (PVP) layer was coated on a silica nanosphere to protect the surface from NaOH attacking by forming hydrogen bonds (Figure 6A).^[^ ^86^ ^]^ As a result, the spherical shape was maintained; meanwhile the OH^−^ ions penetrated through the surface to etch the interior, eventually forming a hollow nanosphere with a porous shell.

Protective agents and etching agents are necessary for maintaining the original morphology and selectively removing the interiors of the templates, respectively. Zhang et al. adopted mesoporous organosilica as the protecting ligand and 1,2‐bis(trimethoxysilyl)ethane (BTME) as the etching agent to synthesize polystyrene sulfonic acid resins (PS‐SO_3_H)@mesosilicas double‐shelled nanostructures (Figure 6B).^[^ ^87^ ^]^ By using surfactant, PS spheres with a silica coating were treated with BTME for preparing PS@mesosilica yolk–shell nanostructures. After removing the surfactants by HCl, PS@mesosilica yolk–shell nanostructures were sulfonated to generate PS‐SO_3_H@mesosilicas double‐shelled nanostructures.

### 2.5 Template contraction and transformation

**FIGURE 7:**
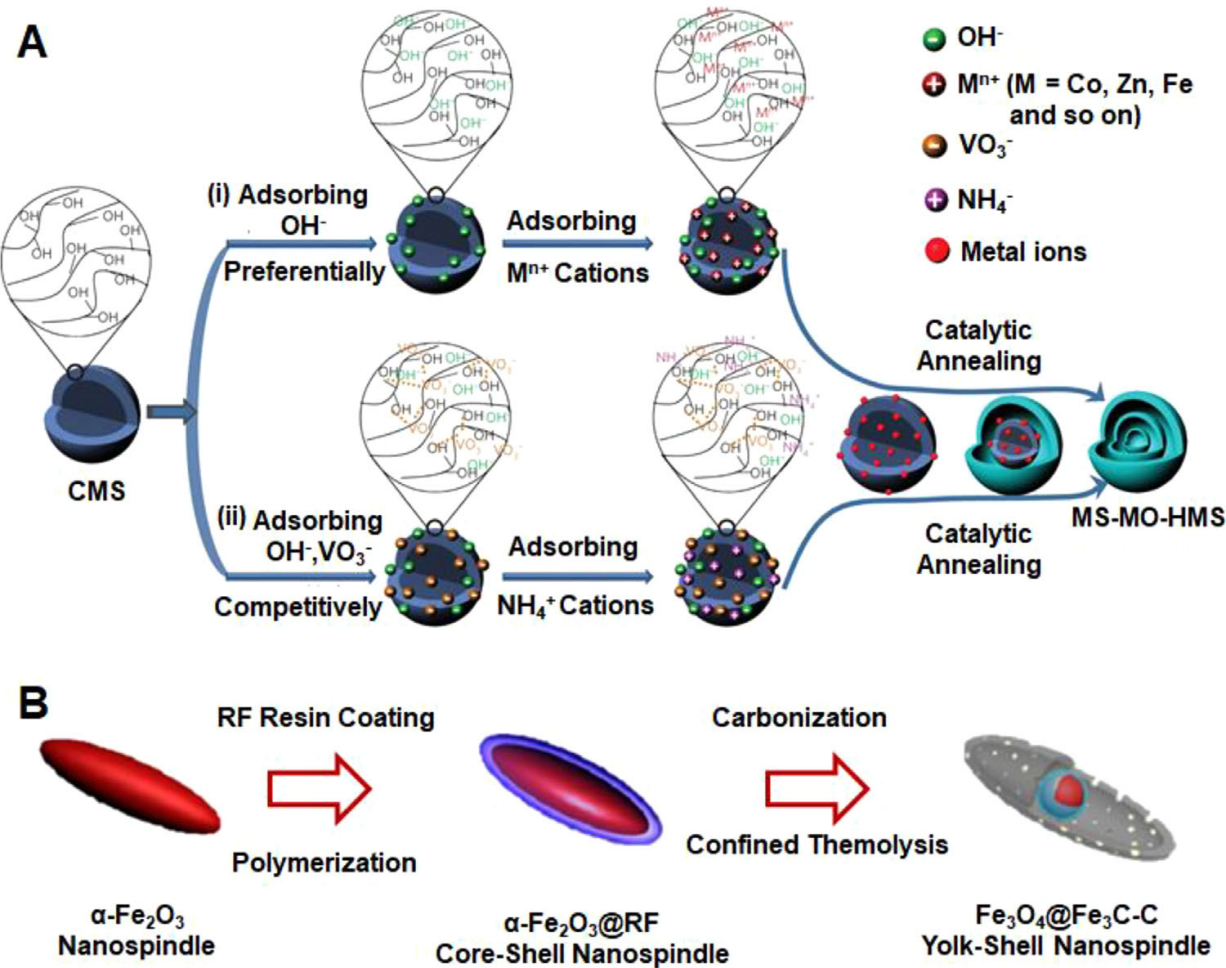
(A) The preparation of multi‐shelled V_2_O_5_ hollow microspheres. Reproduced with permission.^[^ ^90^ ^]^ Copyright 2016, Royal Society of Chemistry. (B) The evolution of yolk–shell Fe_3_O_4_@Fe_3_C‐C nanospindles. Reproduced with permission.^[^ ^91^ ^]^ Copyright 2015, American Chemical Society

Scores of studies have shown that hybrid materials can experience a structural evolution at a high temperature due to the large heterogenous shrinkage in volume; as a result, hollow nanostructures can be created by this thermal‐induced contraction effect.^[^ ^88^ ^]^ Wang et al. proposed a strategy combining the competitive anion adsorption and the catalytic combustion processes to synthesize diverse metal oxide multi‐shelled microspheres.^[^ ^89^, ^90^ ^]^ The formation mechanism is demonstrated in Figure 7A by taking V_2_O_5_ as an example. Since the electrostatic repulsive energy between the negatively charged carbonaceous microspheres (CMSs) and the VO_3_^–^ anions was much smaller than the coordination bond energy between ─OH groups and VO_3_^–^ anions, the CMS templates with abundant ─C─O─C, ─C═O, and ─OH groups preferentially absorbed ions containing similar elements such as OH^–^ or other oxygen‐containing species. The negatively charged CMSs then attracted cations through the electrostatic interactions. Moreover, the V‐based ions can also penetrate into CMSs during the pretreatment by water or NaOH. The synthesis ended with a catalytic annealing step, where the adsorbed V‐based ions served as a catalyst to accelerate the thermal removal of the CMS templates, forming multi‐shelled V_2_O_5_ hollow microspheres. Similarly, MnO_2_, MoO_3_, Cr_2_O_3_, and WO_3_ multi‐shelled hollow microspheres were also prepared using the approach, demonstrating its synthetic versatility.

Another method to induce structural evolution thus creating internal voids is by shrinking the core size, as proposed by Zhang et al. in Figure 7B.^[^ ^91^ ^]^ A resorcinol‐formaldehyde (RF) resin layer was conformably coated on the pre‐formed α‐Fe_2_O_3_ nanospindles. Then the α‐Fe_2_O_3_@RF core–shell nanospindles were in situ annealed in N_2_ at 550°C, which caused the partial “escape” of the core, forming a Fe_3_O_4_@Fe_3_C‐C yolk–shell nanostructure.

## 3 SELF‐TEMPLATING DESIGN OF MICRO/NANOARCHITECTURES

The self‐templating strategy has demonstrated its synthetic versatility in the feasible design of diverse micro/nanoscale architectures, as introduced in the previous section. Extensive studies have shown that the microstructures and morphologies of the products are significantly influenced by the self‐templating synthetic mechanism. Therefore, this section discusses the design of complex micro/nanostructures by self‐templating methods as classified by their configurations, namely surface‐textured solid structures, core–shell structures, yolk–shell structures, single‐shelled hollow structures, multi‐shelled structures, and nanoframes. Comparably, hierarchical micro/nanostructures have their own merits in constructing high‐performance energy storage materials, as listed in Table 2.

**TABLE 2:** The merits of different hierarchical micro/nanostructures in EES applications

Merits					
Hierarchical micro/nanostructures	Specific surface area	Ion diffusion	Gravimetric energy density	Volumetric energy density	Sulfur loading
Surface‐textured solid structures	Low	Low	Low	High	Low
Core–shell structures	Low	Low	Low	High	Low
Yolk–shell structures	Medium	Medium	Medium	Medium	Medium
Single‐shelled hollow structures	High	High	High	Low	High
Multi‐shelled structures	High	High	High	Low	High
Nanoframes	High	High	High	Low	High

### 3.1 Surface‐textured solid structures

**FIGURE 8:**
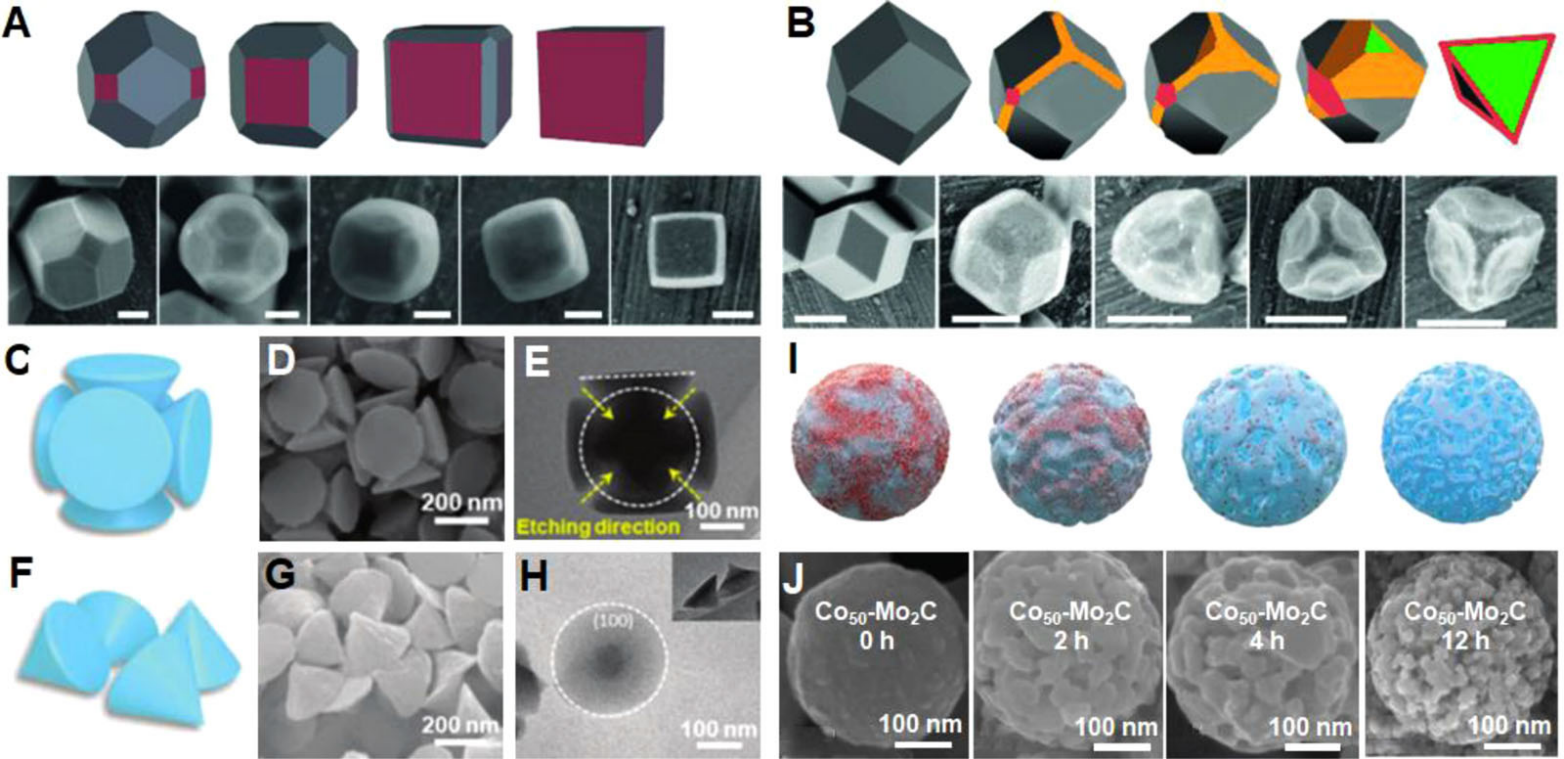
Schematic diagrams and corresponding FESEM images showing the morphology control by adjusting the pH of etchant solution. The rhombic dodecahedral ZIF crystals were derived to (A) cubes and (B) tetrahedrons with etchant solution of pH 3 and pH 3.5, respectively. Scale bar: (A) 100 nm and (B) 500 nm. Reproduced with permission.^[^ ^92^ ^]^ Copyright 2015, Wiley‐VCH. (C–E) Schematic, SEM, and TEM images of NCPs etched for 0.5 h. (F–H) Schematic, SEM, and TEM images of NCPs etched for 1 h. Reproduced with permission.^[^ ^93^ ^]^ Copyright 2020, Royal Society of Chemistry. (I) Schematic and (J) SEM images showing the structural evolution of a Co‐doped Mo_2_C nanosphere against the etching time. Reproduced with permission.^[^ ^94^ ^]^ Copyright 2020, Wiley‐VCH

Solid nanostructures having well‐defined external geometries were considered as a type of energy storage materials; however, they normally demonstrated inferior performances due to the low specific surface area. Therefore, efforts were devoted to constructing surface textures to enlarge the surface area. Avci et al. adopted a wet etching method to re‐shape the zeolitic imidazolate framework (ZIF) nanocrystals.^[^ ^92^ ^]^ It was found that the etching of colloidal ZIFs was anisotropic and surface‐selective. In addition, the crystal topology was simply tuned by adjusting the pH of xylenol orange solution. As the result, the ZIF crystals were intentionally re‐shaped into cubes, tetrahedrons, and hollow boxes by choosing the etchant solution with different pH values (Figure 8A,B). Xu et al. also synthesized Ni‐Co Prussian blue (NCP) analogs with designable nanostructures using the site‐selective and anisotropic etching.^[^ ^93^ ^]^ The chemical etching preferentially started at the corners and edges, gradually proceeded toward the center of NCP cubes. Depending on the etching conditions, complex NCP architectures, including nanocages, nanocubes, nanocones, and chamfer nanocubes were intentionally prepared, enabling the control of morphology with ease (Figure 8C–H).

Ordered porous structures have exhibited superior energy storage capabilities. Metal–organic frameworks (MOFs) as typical porous materials have been widely used as the sacrificial templates for deriving carbon and/or metal/metal oxide‐based micro/nanostructures. Ma et al. synthesized Co‐doped β‐Mo_2_C nanospheres with porous structure and hierarchical surface by means of a combined hydrothermal‐carburization procedure followed by an etching treatment in acid solution.^[^ ^94^ ^]^ Figure 8I shows the evolution of surface morphology of a Co‐Mo_2_C nanosphere against etching time. The original nanosphere possessed a rough surface without any pores, while porous structures were gradually formed on the surface as the etching progressed, which can be attributed to the leaching of Co out of the composite. As a result, a hierarchically porous nanosphere was produced with the etching time of 12 h, as shown in Figure 8J. Yamauchi and co‐workers synthesized MOF networks by using two‐dimensional (2D) double metal hydroxide (LDH) templates through a confined growth method. The MOF networks were then converted to honeycomb‐like microporous carbon flakes by pyrolysis in N_2_. Specifically, the microporous carbon flakes exhibited adjustable properties owing to the tunable metal compositions in the precursors.^[^ ^95^, ^96^ ^]^

### 3.2 Core–shell structures

**FIGURE 9:**
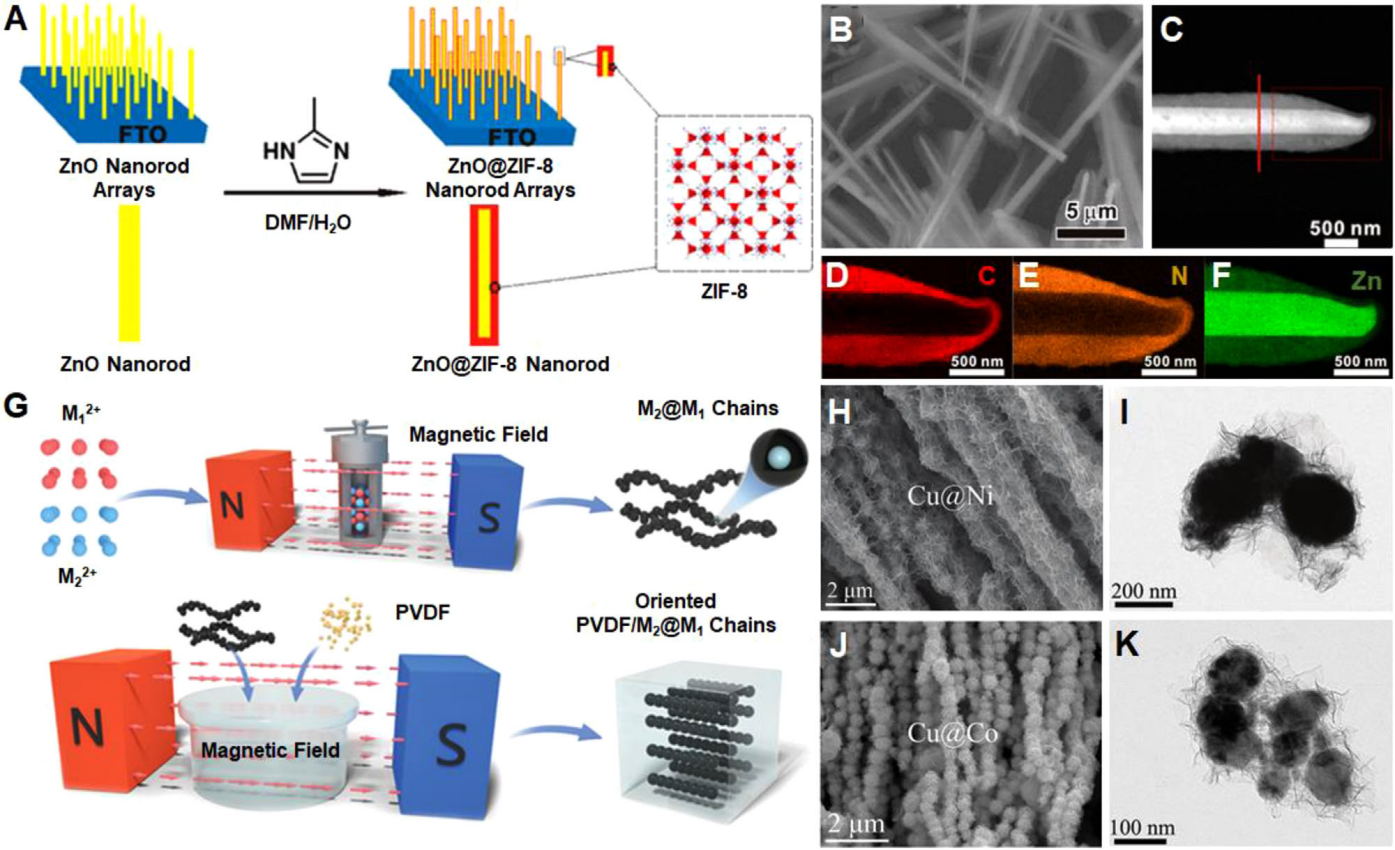
(A) The preparation of the nanorod arrays with ZnO core and ZIF‐8 shell. (B) SEM, (C) STEM image, and (D–F) the elemental maps of ZnO@ZIF‐8 nanorods. Reproduced with permission.^[^ ^99^ ^]^ Copyright 2013, American Chemical Society. (G) The growth of aligned 1D core–shell bimetallic nanostructures under magnetic field. (H) SEM and (I) TEM images of core–shell Cu@Ni chains. (J) SEM and (K) TEM images of core–shell Cu@Co chains. Reproduced with permission.^[^ ^100^ ^]^ Copyright 2020, Wiley‐VCH

Core–shell structures refer to the configuration of the central core directly encapsulated by another shell material. The core–shell nanostructures not only inherit the characteristics from both the core and shell materials, but also gain novel properties associated with the interface.^[^ ^97^, ^98^ ^]^ Chemical etching has been widely adopted to synthesize core–shell heterostructures. For instance, Zhan et al. successfully prepared freestanding ZnO@ZIF‐8 nanorods by in situ growing ZIF‐8 on ZnO templates (Figure 9A).^[^ ^99^ ^]^ The Zn^2+^ ions were released from ZnO nanorods as a result of etching by 2‐methylimidazol, then coordinated with the 2‐methylimidazol ligands to form ZIF‐8 coating layer (Figure 9B–F). The core–shell heterostructures were facilely controlled by balancing the dissolution of ZnO templates and the growth of ZIF‐8.

Galvanic replacement is normally employed to construct core–shell bimetallic nanostructures. For example, as schematically demonstrated in Figure 9G, Zhao et al. fabricated one‐dimensional (1D) core–shell Cu@Ni (Figure 9H,I) and Cu@Co (Figure 9J,K) bimetallic magnetic chains through a magnetic field assisted galvanic replacement.^[^ ^100^ ^]^ In the polyol solution, the generated radicals served as reducing agents. Since Cu species had a higher standard potential compared with Ni and Co species, Cu nanoparticles were first obtained forming a Cu core. Ni and Co nanoparticles were subsequently reduced and coated on the Cu cores, resulting in core–shell Cu@Ni and Cu@Co composite chains with the assisted alignment by the magnetic field. The facile fabrication method was also applicable to construct other 1D core–shell bimetallic chains composed of various noble metals.

### 3.3 Yolk–shell structures

**FIGURE 10:**
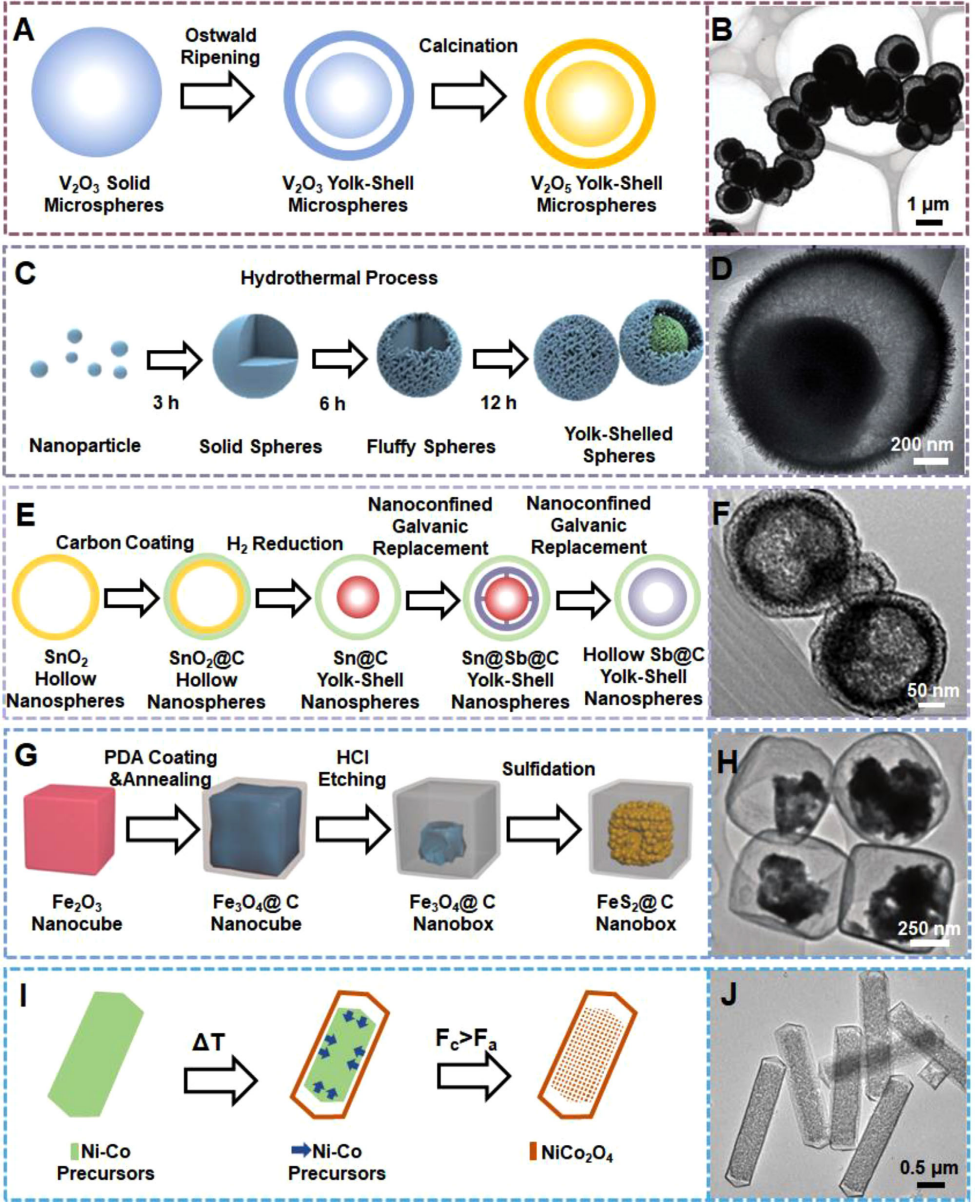
(A) The synthesis and (B) TEM image of V_2_O_5_ microspheres with yolk–shell structures. Reproduced with permission.^[^ ^105^ ^]^ Copyright 2011, Royal Society of Chemistry. (C) The hydrothermal synthesis and (D) TEM image of yolk–shell WVO*_x_* nanoparticles. Reproduced with permission.^[^ ^106^ ^]^ Copyright 2021, Royal Society of Chemistry. (E) The formation and (F) TEM image of the Sb@C nanospheres derived from SnO_2_ hollow nanospheres. Reproduced with permission.^[^ ^107^ ^]^ Copyright 2017, American Chemical Society. (G) The production of FeS_2_@C yolk–shell nanoboxes by chemical etching and sulfidation. (H) TEM image of the FeS_2_@C yolk–shell nanoboxes. Reproduced with permission.^[^ ^108^ ^]^ Copyright 2017, Royal Society of Chemistry. (I) Schematic of the generation of Ni‐Co mixed oxide nanoprisms by thermal contraction. (J) TEM image of the as‐synthesized NiCo_2_O_4_ yolk–shell nanoprisms. Reproduced with permission.^[^ ^109^ ^]^ Copyright 2015, Wiley‐VCH

Different from the close contact in core–shell structure, there are abundant voids thereby creating clear space between the two components in a yolk–shell structure. The unique hollow structure can not only protect the inner yolk from aggregation and deactivation, but also offer a pathway for efficient mass transfer.^[^ ^101^, ^102^, ^103^, ^104^ ^]^ Yolk–shell structures have been successfully synthesized by various self‐templating strategies. For example, yolk–shell V_2_O_5_ microspheres were synthesized in a solvothermal system.^[^ ^105^ ^]^ As illustrated in Figure 10A, solid intermediate V_2_O_3_ microspheres were first obtained within a short solvothermal reaction period, which were then transformed into a yolk–shell structure through an Ostwald ripening process. Finally, the V_2_O_3_ yolk–shell microspheres were further oxidized at a higher calcination temperature, forming crystallized V_2_O_5_ yolk–shell microspheres (Figure 10B).

Shi et al. synthesized a series of yolk–shell transition metal oxides (MnVO*_x_*, FeVO*_x_*, CoVO*_x_*, and ZnVO*_x_*) via the combination of the Ostwald ripening and the Kirkendall effect.^[^ ^106^ ^]^ As illustrated in Figure 10C, WVO*_x_* solid nanoparticles were first obtained within short‐time hydrothermal process, the surface of which became rougher with the prolongation of the reaction time. Further hydrothermal treatment resulted in the generation of yolk–shell nanospheres (Figure 10D) owing to the interdiffusion and re‐crystallization by combination of Ostwald ripening and Kirkendall effect. Zhu and co‐workers fabricated hollow Sb@C yolk–shell nanospheres via a self‐templating route.^[^ ^107^ ^]^ As schematically illustrated in Figure 10E, uniform SnO_2_ nanospheres with Ostwald ripening‐induced hollow structures were first synthesized by hydrothermal treatment and coated with polysaccharide, then functioned as the templates for subsequent reactions. The hybrids were annealed in H_2_, transforming carbon‐rich polysaccharide into a carbon shell and SnO_2_ into a Sn core, and Sn@C yolk–shell structures with internal spaces provided by the hollow SnO_2_ templates were eventually achieved. The as‐prepared Sn@C yolk–shell nanospheres were employed as precursors for in situ synthesizing Sb@C yolk–shell hollow nanospheres with the help of a confined galvanic replacement between Sn and Sb^3+^, together with the generation of hollow cavities as a result of the interdiffusion between Sn and Sb (Figure 10F).

The design of FeS_2_@carbon (FeS_2_@C) with a unique yolk–shell nanoarchitecture was achieved by a chemical etching coupled with a sulfidation strategy, as reported by Liu et al. in 2017 (Figure 10G).^[^ ^108^ ^]^ First, a polydopamine (PDA) layer was homogeneously coated on Fe_2_O_3_ nanocubes and subsequently calcined in Ar, forming Fe_3_O_4_@C nanocubes as a result of the polymer pyrolysis. The Fe_3_O_4_@C nanocubes were then treated with HCl and the Fe_3_O_4_ core was partially removed, leading to yolk–shell nanoboxes. Finally, a sulfidation‐in‐nanobox approach was adopted to transform Fe_3_O_4_ into FeS_2_, obtaining FeS_2_@C yolk–shell nanoboxes as shown in Figure 10H.

Yu et al. adopted a fast thermal‐induced contraction strategy to prepare yolk–shell nanoprisms with mesoporous structures.^[^ ^109^ ^]^ Initially, monodisperse Ni‐Co precursor nanoparticles with tetragonal prism‐like architecture were obtained through a co‐precipitation method using PVP as the structure‐stabilizer and then annealed in air to form oxides, forming a yolk–shell structure due to the heterogeneous contraction in volume (Figure 10I,J).

### 3.4 Single‐shelled hollow structures

Single‐shelled hollow structures possess intriguing structural features including a kinetically favorable open frame, a high surface area, superior surface permeability, and a low density owing to its well‐defined cavities.^[^ ^19^, ^110^ ^]^ A myriad of self‐templating strategies were implemented for synthesizing single‐shelled hollow architectures with tunable compositions.

**FIGURE 11:**
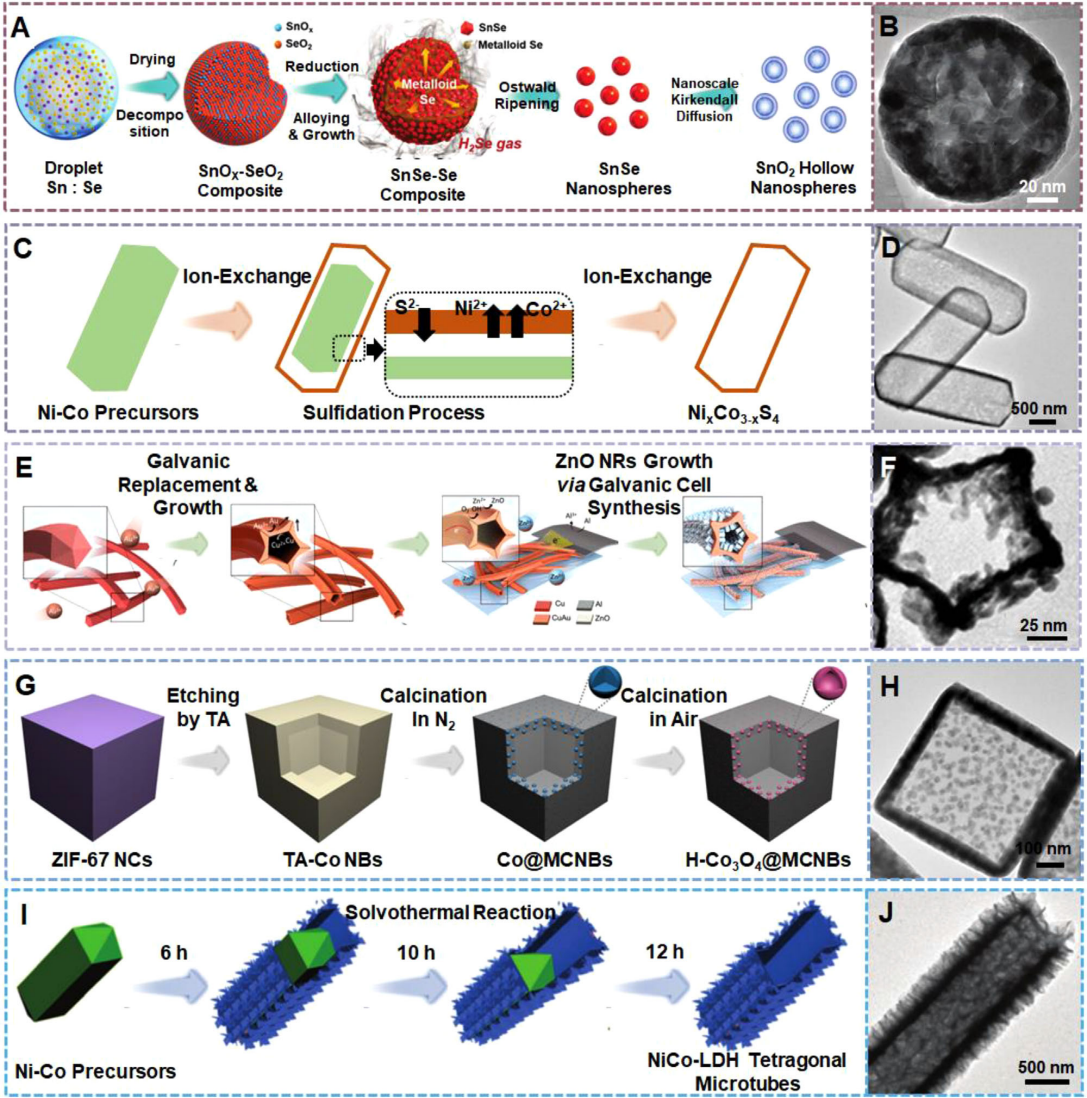
(A) The preparation and (B) TEM observation of SnO_2_ hollow nanospheres. Reproduced with permission.^[^ ^111^ ^]^ Copyright 2018, Royal Society of Chemistry. (C) The evolution and (D) TEM image of Ni*_x_* Co_3−_ *_x_* S_4_ hollow nanoprisms. Reproduced with permission.^[^ ^112^ ^]^ Copyright 2014, Wiley‐VCH. (E) The preparation of CuAu‐ZnO hollow nanostartubes. (F) TEM image of a CuAu nanostartube. Reproduced with permission.^[^ ^113^ ^]^ Copyright 2018, American Chemical Society. (G) The formation of H‐Co_3_O_4_@MCNBs by chemical etching and calcination. (H) TEM image of a H‐Co@MCNB. Reproduced with permission.^[^ ^114^ ^]^ Copyright 2020, Wiley‐VCH. (I) The structural evolution from the original solid prism to the final hollow tube in a solvothermal reaction. (J) TEM image of a NiCo‐LDH tetragonal microtube. Reproduced with permission.^[^ ^115^ ^]^ Copyright 2016, Royal Society of Chemistry

Ostwald ripening and Kirkendall effect were applied in the preparation of SnO_2_ single‐shelled hollow nanospheres by Park et al.^[^ ^111^ ^]^ As schematically shown in Figure 11A, SnSe nanospheres were formed by the pyrolysis of a Se‐rich spray solution following the Ostwald ripening kinetics. In the final step, SnSe nanospheres were calcinated in air to produce SnO_2_ hollow nanospheres (Figure 11B) as a result of the Kirkendall diffusion.

Yu et al. fabricated ternary Ni*_x_* Co_3−_ *_x_* S_4_ hollow nanoprisms with defined hollow interiors by a sulfuration reaction between the Ni‐Co acetate hydroxide precursors and the thioacetamide (TAA) sulfur source.^[^ ^112^ ^]^ As shown in Figure 11C, the released S^2–^ ions reacted with the metal ions forming a thin layer of Ni‐Co sulfides. Direct chemical reaction was hindered by the Ni‐Co sulfides barrier so that further reactions can only depend on the inward and outward ion diffusion through the barrier, thus forming a hollow interior (Figure 11D).

Tan et al. fabricated hollow stellated CuAu‐ZnO nanostartubes through a two‐step galvanic reaction.^[^ ^113^ ^]^ As demonstrated in Figure 11E, pentagonal Cu nanowires were synthesized and utilized as the templates. Au^3+^ ions were added to induce a facet‐specific galvanic replacement to realize simultaneous stellation and cavitation of the multiply twinned CuAu nanostartubes (Figure 11F). Finally, ZnO nanorods were grown on the walls of the CuAu nanostartubes through a galvanic cell reaction, forming CuAu‐ZnO nanostartubes.

Huang et al. employed ZIF‐67 nanocubes as the templates to synthesize well‐defined hybrid hollow nanostructures consisting of carbon nanoboxes embedded with ultrafine Co_3_O_4_ hollow nanoparticles by utilizing a combined chemical etching and annealing method,^[^ ^114^ ^]^ as shown in Figure 11G. Tannic acid (TA) was employed as the etchant to react with the pre‐formed ZIF‐67 nanocubes, resulting in TA‐Co hollow nanoboxes. The subsequent annealing treatment in N_2_ enabled the pyrolysis of TA, producing mesoporous carbon nanocubes as well as reducing Co^2+^ to Co nanoparticles. The embedded Co nanoparticles were finally oxidized in air, forming hollow Co_3_O_4_ nanoparticles embedded in mesoporous carbon nanoboxes (Figure 11H).

The same group also produced 1D hybrid hollow nanostructures by using a solvothermal method.^[^ ^115^ ^]^ As shown in Figure 11I, metal acetate hydroxide solid microprisms were first produced at an early solvothermal reaction stage, then functioned as the self‐supporting templates to be transformed into NiCo‐layered double hydroxide (NiCo‐LDH) microtubes decorated with sheet‐like subunits. The hierarchical shell continued to grow as the solvothermal reaction progressed, while the inner core started to shrink, finally resulting in NiCo‐LDH hollow tetragonal microtubes (Figure 11J).

### 3.5 Multi‐shelled structures

**FIGURE 12:**
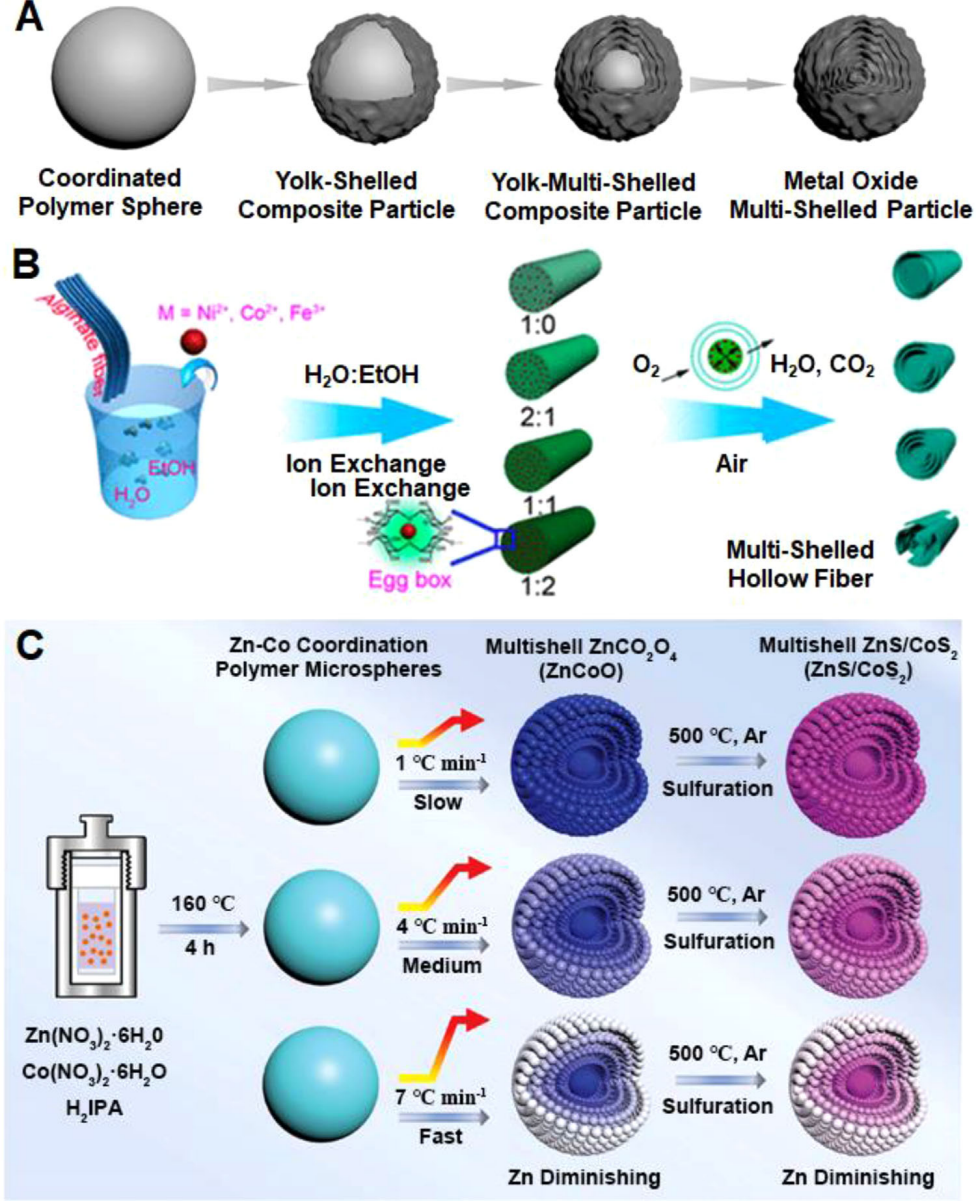
(A) The preparation of Ni‐Co oxide multi‐shelled particles. Reproduced with permission.^[^ ^119^ ^]^ Copyright 2017, Wiley‐VCH. (B) The formation of different multi‐shelled microfibers by tuning the H_2_O/EtOH ratio. Reproduced with permission.^[^ ^120^ ^]^ Copyright 2017, American Chemical Society. (C) The synthesis of ZnS/CoS_2_ multi‐shelled microspheres with slow, medium, and fast heating rates, and the subsequent sulfuration treatments in Ar. Reproduced with permission.^[^ ^121^ ^]^ Copyright 2020, Wiley‐VCH

Nanostructures with multiple shells can overcome the drawback of low packing density in single‐shelled hollow structures.^[^ ^116^, ^117^ ^]^ In addition, the multiple shells offer a tunable composition in different layers of the structure, enabling synergetic effect thus improving the overall performance.^[^ ^23^, ^118^ ^]^ Guan et al. developed a versatile thermal oxidation strategy for preparing ternary and quaternary metal oxide nanostructures with multi‐shelled hollow configuration.^[^ ^119^ ^]^ Figure 12A illustrates the formation of multi‐shelled hollow metal oxide nanostructures under heat treatment. While the coordinated polymer solid nanoparticle was thermally decomposed, the weight loss induced large volume shrinkage thus leading to the spallation of the oxide layer and the formation of a yolk–shell structure. As the calcination temperature increased, the heterogeneous contraction became more severe, forming yolk–multi‐shelled structure and ultimately multi‐shelled hollow nanoparticle.

Intriguingly, Sun et al. demonstrated the control over shell numbers of transition‐metal oxides multi‐shelled hollow fibers by adjusting the loading and diffusion length of metal ions in polymer precursors.^[^ ^120^ ^]^ The adsorption of metal ions and their diffusion depth in the alginate fiber were found to be controlled by the wettability of the fiber surface. The ethanol solvent successfully lowered the surface tension thus improving the adsorption of metal ions; as the result, multiple shells were generated during the oxidation of the alginate fiber under heat treatment in air. As displayed in Figure 12B, NiO hollow fibers with single‐shell, double‐shell, and triple‐shell configurations were respectively prepared by changing the H_2_O/ethanol ratio from 1:0 to 1:1. However, broken shells were observed when further increasing the volume ratio to 2:1. The versatile strategy was applicable to diverse transition metal oxide hollow fibers, that is, Co_3_O_4_ and Fe_2_O_3_ multi‐shelled hollow fibers were also synthesized by this strategy.

The element distribution from core to shell in multi‐shelled spheres is possibly adjusted by controlling the heating ramp rate and the diffusion coefficient of different elements.^[^ ^121^ ^]^ As shown in Figure 12C, Zn‐Co coordination polymer microspheres as the templating precursors were annealed in air for synthesizing ZnCo_2_O_4_ multi‐shelled microspheres via a heterogeneous shrinkage during the calcination process. It was found that the elemental composition distribution was determined by the thermal diffusion of Zn and Co during the solidification, which mainly depended on the heating temperature, ramp rate, and diffusion activation energy. At the same heating temperature, a higher ramp rate promoted the diffusion of Zn since it has larger diffusion coefficient than Co, resulting in a gradient distribution of Zn in the ZnCo_2_O_4_ microspheres. Further sulfuration in Ar produced ZnS/CoS_2_ multi‐shelled microspheres with unaltered elemental gradient distribution.

### 3.6 Nanoframes

**FIGURE 13:**
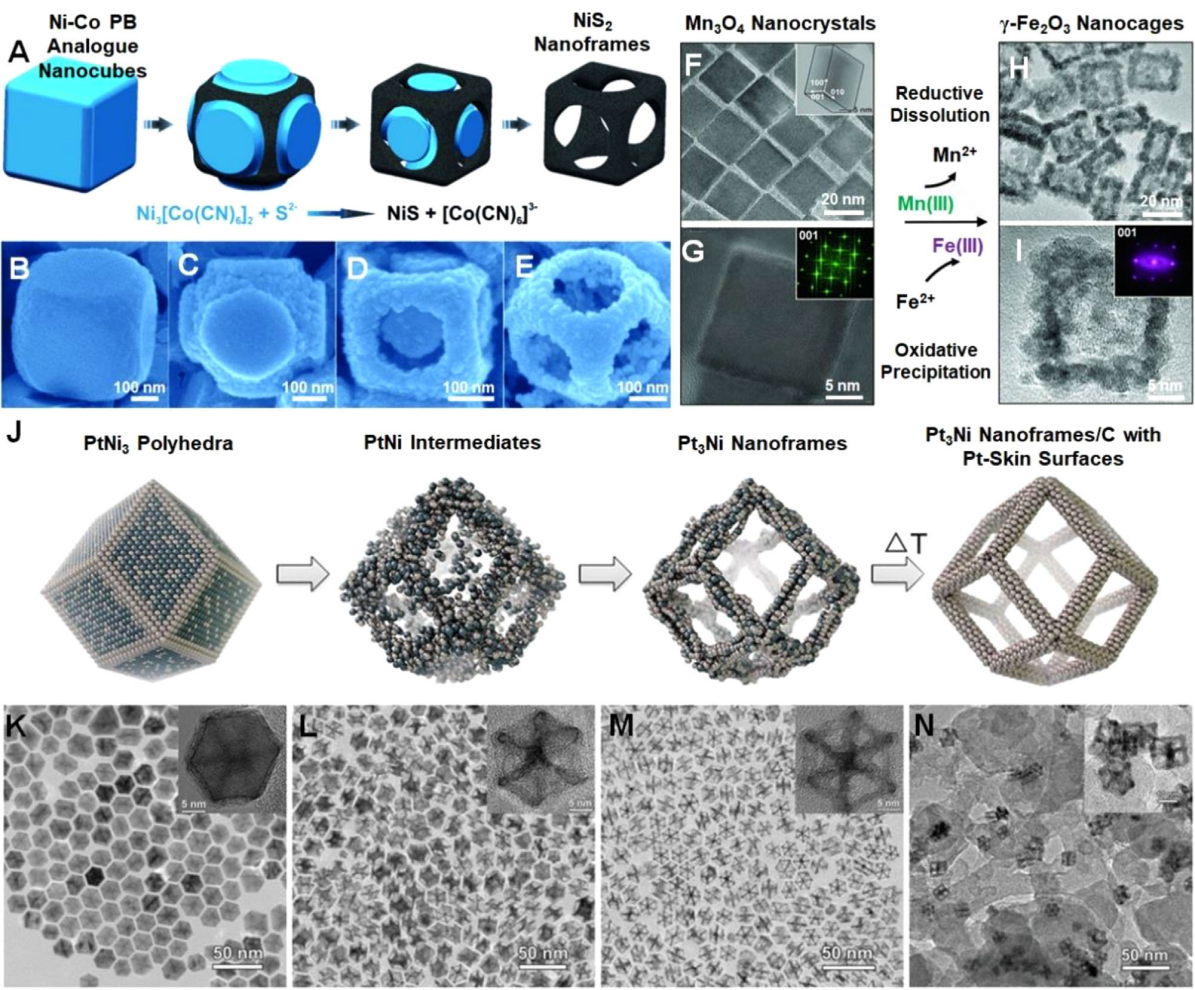
(A) Schematic reaction process for the formation of NiS_2_ nanoframes. SEM images of the Ni‐Co PBA nanocube etched for (B) 0 h, (C) 0.5 h, (D) 2 h, and (E) 6 h. Reproduced with permission.^[^ ^122^ ^]^ Copyright 2015, Wiley. (F) TEM and (G) HRTEM images of Mn_3_O_4_ nanocages. (H) TEM and (I) HRTEM images of the obtained γ‐Fe_2_O_3_ nanocages. Reproduced with permission.^[^ ^123^ ^]^ Copyright 2013, American Association for the Advancement of Science. (J) Schematic diagram and the corresponding (K–N) TEM images of multimetallic nanoframes via chemical etching. Reproduced with permission.^[^ ^124^ ^]^ Copyright 2014, American Association for the Advancement of Science

Nanoframe is a special class of 3D structure with unique open architecture and well‐defined surface composition. The synthesis of nanoframes has been explored extensively, and various self‐templating strategies have been implemented to prepare nanoframes. For instance, Yu et al. utilized Ni‐Co Prussian blue analog (PBA) as the precursor to prepare NiS nanoframes via a chemical etching/anion exchange reaction with S^2–^ ions,^[^ ^122^ ^]^ as illustrated in Figure 13A. The chemical etching/anion exchange was anisotropic because of the special structure of the PBA nanocubes (TEM in Figure 13B), of which the edges with high curvature were rougher so as to provide more reactive sites to enhance the chemical reactions. Hence, a thin NiS shell was preferentially grown on the edges of the templating nanocubes (Figure 13C). The lateral sides of the Ni‐Co PBA nanocubes were further exposed to Na_2_S solution, so the anion exchange occurred on the middle plane surfaces, generating voids at the interface due to the unequal diffusion of Ni^2+^ and S^2–^ ions. The continuous anion exchange reaction enabled the generation of NiS on the nanocube edges, whereas the central parts were etched away (Figure 13D), eventually producing well‐defined NiS nanoframes (Figure 13E). Oh et al. successfully synthesized various metal oxide nanoframes by means of galvanic replacement reactions.^[^ ^123^ ^]^ For example, γ‐Fe_2_O_3_ nanocages were produced through a galvanic reaction between iron(II) and Mn_3_O_4_ nanoparticles. The continuous reductive dissolution of the cores of the Mn_3_O_4_ nanocrystals and the oxidative precipitation of γ‐Fe_2_O_3_ resulted in the generation of Mn_3_O_4_/γ‐Fe_2_O_3_ hollow nanoboxes (Figure 13F–I), which were eventually transformed into γ‐Fe_2_O_3_ nanocages with clear open architectures. In addition to metal oxide nanoframes, highly crystalline bimetallic nanoframes were obtained by Stamenkovic and co‐workers.^[^ ^124^ ^]^ As shown in Figure 13J–N, the templating PtNi_3_ polyhedra were gradually transformed into Pt_3_Ni nanoframes by interior erosion. Subsequent annealing in Ar further smoothened the surface of the nanoframes.

## 4 ENERGY STORAGE APPLICATIONS

As elaborately discussed in previous sections, hierarchical micro/nanostructures with tailorable configurations can be effectively designed and achieved by the self‐templating strategies. Since the morphology and structure of a material have great impact on their electrochemical performances, this section mainly discusses the merits of hierarchical micro/nanostructures for constructing diverse high‐performance EES devices, including supercapacitors (SCs), lithium‐ion batteries (LIBs), lithium–sulfur batteries (LSBs), sodium‐ion batteries (SIBs), potassium‐ion batteries (PIBs), lithium–air batteries (LABs) and zinc–air batteries (ZABs).

### 4.1 Supercapacitors

**FIGURE 14:**
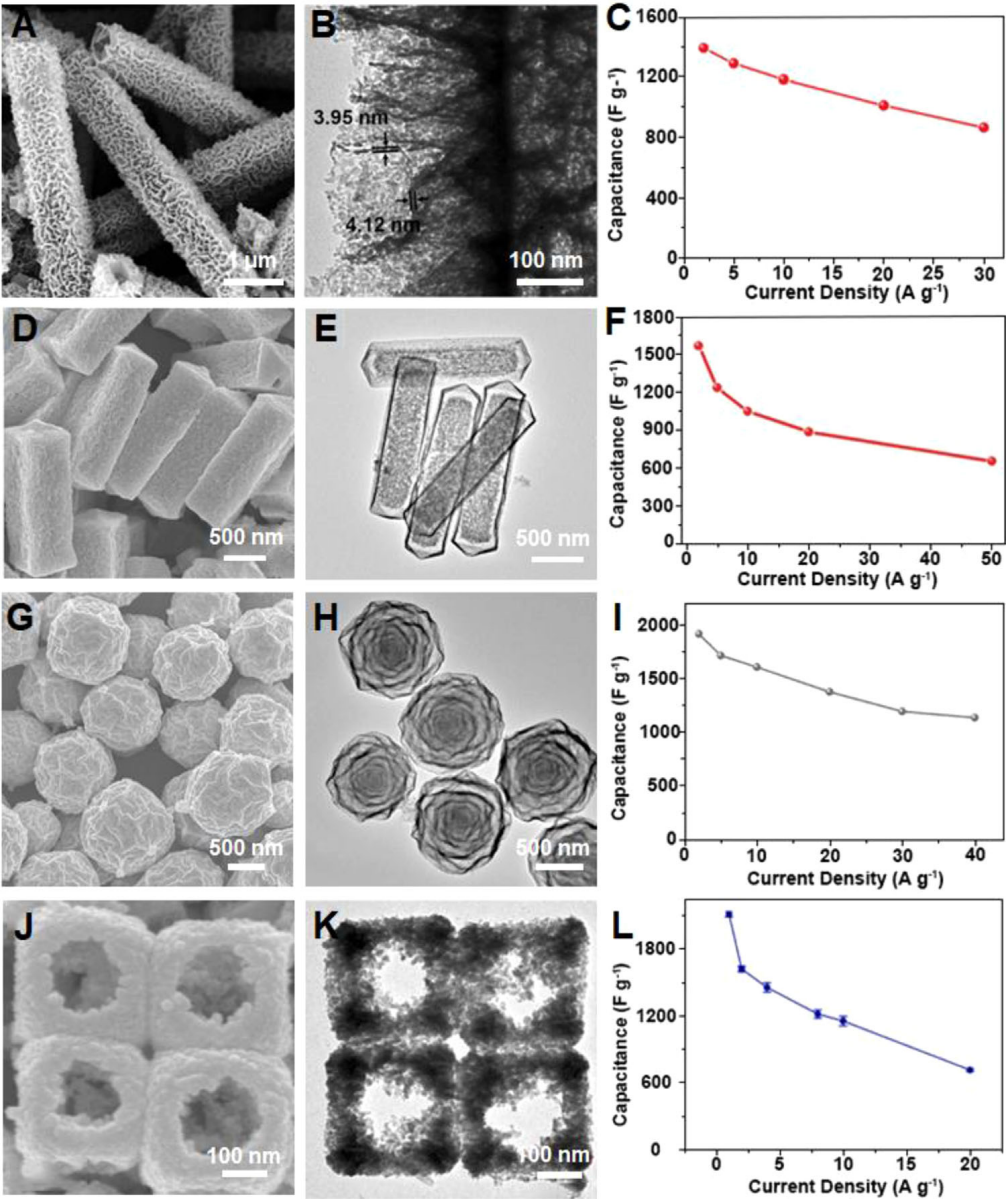
(A) SEM image, (B) TEM image, and (C) rate performance of hierarchical NiCo_2_O_4_ tetragonal microtubes. Reproduced with permission.^[^ ^115^ ^]^ Copyright 2016, Royal Society of Chemistry. (D) SEM image, (E) TEM image, and (F) rate performance of Ni_0.37_Co oxides nanoprimes. Reproduced with permission.^[^ ^109^ ^]^ Copyright 2015, Wiley‐VCH. (G) SEM image, (H) TEM image, and (I) rate performance of multi‐shelled Ni‐Co oxide nanoparticles. Reproduced with permission.^[^ ^119^ ^]^ Copyright 2017, Wiley‐VCH. (J) SEM image, (K) TEM image, and (L) rate performance of NiS nanoframes. Reproduced with permission.^[^ ^122^ ^]^ Copyright 2015, Wiley‐VCH

As one of the most widely used EES devices, SCs possess intriguing features of excellent charge/discharge characteristics and a long servicing lifespan.^[^ ^125^, ^126^, ^127^, ^128^ ^]^ Especially, the hybrid supercapacitors combine the merits of batteries (high energy density) and SCs (high power capability) simultaneously.^[^ ^129^, ^130^, ^131^ ^]^ However, the battery‐type electrodes of the hybrid supercapacitors normally exhibit sluggish kinetics and unsatisfactory cycle life owing to the long diffusion pathway and the large volume change.^[^ ^132^, ^133^ ^]^ An effective strategy to tackle the severe issues is to construct electrode materials with hierarchical micro/nanostructures by shortening the diffusion distance and alleviating the volume expansion so as to improve the EES performances.^[^ ^134^, ^135^ ^]^ For example, hierarchical tetragonal microtubes assembled by mesoporous NiCo_2_O_4_ nanosheets (Figure 14A,B) were employed as the battery‐type electrodes for hybrid supercapacitors.^[^ ^115^ ^]^ The unique hierarchical architecture offered distinct structural merits. First of all, the assembled nanosheets provided a high surface area, leading to enriched active sites exposed for redox reactions. Besides, the open space between the nanosheets enhanced the electrode‐electrolyte interactions, resulting in an improved capacitance and rate capability. The NiCo_2_O_4_ microtubes provided 1387.9 F g^–1^ at 2 A g^–1^ with 62% retained at 30 A g^–1^ (Figure 14C). Moreover, the cycling performance of the electrode manifested 10.6% capacitance loss after continuous charge/discharge for 12,000 cycles at 10 A g^–1^, attributable to the structural integrity enabled by the hollow interior within the hierarchical microtube.

Yu et al. constructed hybrid supercapacitors based on Ni‐Co oxide electrodes with mesoporous yolk–shell nanostructures (Figure 14D,E).^[^ ^109^ ^]^ 1563 F g^–1^ was delivered by the electrode at 2 A g^–1^ and 651 g^–1^ was retained at 50 A g^–1^ (Figure 14F). The cycling reliability of the electrode was outstanding that the specific capacitance showed only a small loss of 2% after continuous charge/discharge for 15,000 cycles at 10 A g^–1^. Such performance was attributed to rich sites for charge storage and efficient diffusion of ions into the porous nanoprisms. Besides, the yolk–shell hollow features effectively accommodated the volume variation thus enhancing the cycling stability. The same group further improved the electrochemical performance in their subsequent works, where the Ni‐Co oxide nanoparticles‐based electrode was designed with a multi‐shelled onion‐like structure (Figure 14G,H).^[^ ^119^ ^]^ Intriguingly, the multi‐shelled Ni‐Co oxide exhibited 1908 F g^–1^ at 2 A g^–1^ and maintained 1129 F g^–1^ at 40 A g^–1^ (Figure 14I). The electrode was also cyclic tested at 10 A g^–1^ with 93.6% of initial capacitance retained after cycling 20,000 times. TEM observations revealed that the multiple shells were porous with substantial voids, which can accommodate volume changes at nanograin level through minimizing the structural change during charge/discharge cycles, thus enhancing rate capability and cycling stability.

Well‐defined NiS nanoframes (Figure 14J,K) have demonstrated excellent electrochemical properties as SC materials.^[^ ^122^ ^]^ The specific capacitance reached a high value of 2122 F g^–1^ at 1 A g^–1^ (Figure 14L). Even at 20 A g^–1^, the specific capacitance can retain the value of 711 F g^–1^. This was ascribed to the open‐frame and porous structures which enlarged electrode‐electrolyte interfacial surfaces and offered substantial redox sites. Furthermore, the electrode retained 90.8% of initial capacitance after 4000 cycles at 4 A g^–1^, which was attributed to the structural robustness of the nanoframes. Overall, the electrochemical performances of the electrode materials with different hierarchical micro/nanostructures for supercapacitors are compared in Table 3.

**TABLE 3:** Electrochemical performances of different hierarchical micro/nanostructures prepared by self‐templating strategies for supercapacitors

Supercapacitors					
Hierarchical architectures	Electrode materials	Self‐templating synthetic mechanism	Highest reversible capacity	Cycling performance	Ref.
Hierarchical tetragonal microtube	NiCo_2_O_4_	Template contraction and transformation	1387.9 F g^–1^ 2 A g^–1^	89.4% retention, 10 A g^–1^, 12,000 cycles	[115]
Mesoporous yolk–shell nanostructure	NiCo oxide	Ion exchange	1563 F g^–1^ 2 A g^–1^	98% retention, 10 A g^–1^, 15,000 cycles	[109]
Multi‐shelled onion‐like structure	NiCo oxide	Template contraction and transformation	1908 F g^–1^ 2 A g^–1^	93.6% retention, 10 A g^–1^, 20,000 cycles	[119]
Nanoframe	NiS	Ion exchange	2122 F g^–1^ 1 A g^–1^	91.8% retention, 4 A g^–1^, 4000 cycles	[122]
Morning glory‐like porous architecture	NiO/ NiCo_2_O_4_	Chemical etching	378 C g^–1^ 2 A g^–1^	N.A.	[93]
Double‐shelled nanocage	Co_3_O_4_/NiCo_2_O_4_	Chemical etching	972 F g^–1^ 5 A g^–1^	92.5% retention, 10 A g^–1^, 12,000 cycles	[136]
Hollow nanoprism	Ni*_x_* Co_3−_ *_x_* S_4_	Ion exchange	895.2 F g^–1^ 1 A g^–1^	85.7% retention, 5 A g^–1^, 1500 cycles	[112]
Hollow sphere	NiCo_2_O_4_	Template contraction and transformation	1141 F g^–1^ 1 A g^–1^	94.7% retention, 5 A g^–1^, 4000 cycles	[61]
Mesoporous structure	MoO_2_@rGO	Chemical etching	372.5 F g^–1^ 0.1 A g^–1^	96% retention, 5 A g^–1^, 10,000 cycles	[137]
Honeycomb‐like structure	Zn‐ZIF‐C	Template contraction and transformation	221 F g^–1^ 0.5 A g^–1^	100% retention, 4 A g^–1^, 10,000 cycles	[96]

### 4.2 Lithium‐ion batteries

LIBs are the mainstream energy storage system for powering electronic devices and hybrid vehicles, benefitted from their high energy densities, high coulombic efficiencies, and slow self‐discharge properties.^[^ ^138^, ^139^, ^140^, ^141^ ^]^ A LIB typically consists of four components: an anode, an electrolyte, a separator, and a cathode. Metal oxides,^[^ ^142^, ^143^ ^]^ sulfides,^[^ ^144^, ^145^ ^]^ and phosphides^[^ ^146^, ^147^ ^]^ have shown great promises as the anode candidates owing to their high theoretical capacities compared with the commonly used graphite electrodes. Nevertheless, the large volume change upon charge/discharge cycles unavoidably destroys the material integrity, resulting in poor cycling performances.^[^ ^148^, ^149^ ^]^ A viable method to mitigate the severe issues is to develop hierarchical materials that can provide abundant Li^+^ storage sites by enlarging electrode/electrolyte interfacial surface area and accommodate the volume changes by alleviating the cycling‐induced strains.^[^ ^150^ ^]^

**FIGURE 15:**
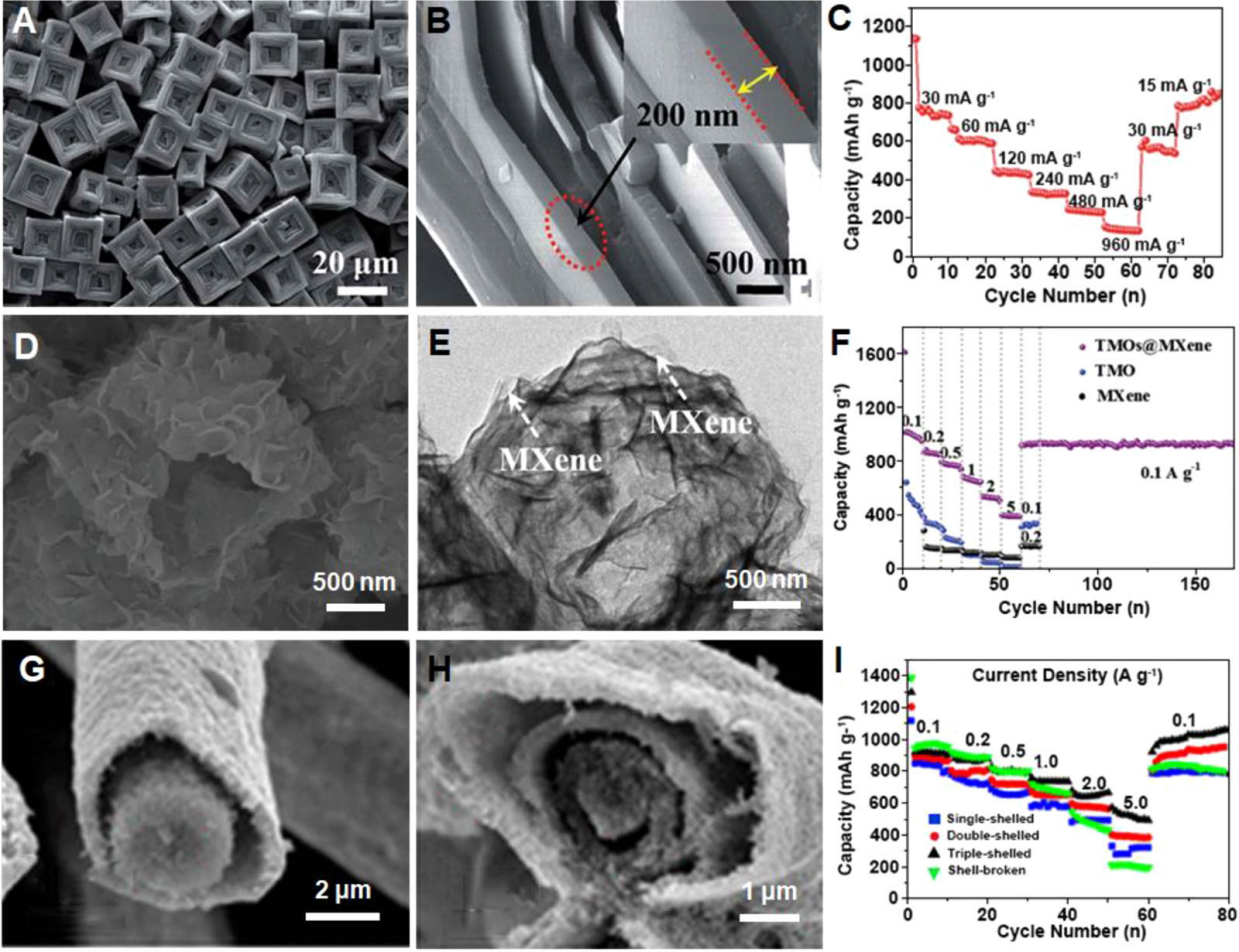
(A,B) SEM images and (C) rate performance of PB microframes with kinked surfaces. Reproduced with permission.^[^ ^151^ ^]^ Copyright 2019, Royal Society of Chemistry. (D) SEM and (E) TEM images of CoO/Co_2_Mo_3_O_8_@MXene hollow polyhedrons. (F) Rate capabilities of TMO, MXene, and CoO/Co_2_Mo_3_O_8_@MXene anodes. Reproduced with permission.^[^ ^152^ ^]^ Copyright 2020, Wiley‐VCH. SEM of (G) single‐shelled and (H) triple‐shelled NiO hollow microfibers. (I) Rate capabilities of NiO anodes with different shell numbers. Reproduced with permission.^[^ ^120^ ^]^ Copyright 2017, American Chemical Society

Hierarchically structured Prussian blue (PB) microframes with kinked surfaces were also explored as anode materials for LIBs (Figure 15A,B).^[^ ^151^ ^]^ The discharge capacities of PB microframes were measured to be 730, 600, 430, and 320 mAh g^–1^ at 30, 60, 120, and 240 mA g^–1^ (Figure 15C), respectively. Specifically, at 960 mA g^–1^, the PB microframes still delivered a high specific capacity of 130 mAh g^–1^. 76.7% of capacity was recovered as the current density dropped back to 30 mA g^–1^, suggesting an excellent rate performance. Furthermore, the PB microframes also exhibited a stable discharge specific capacity, with 548 mAh g^–1^ retained over 550 cycles at 215 mA g^–1^. The unique structure was of significance to enhance the electrochemical performances of PB microframes, where the high crystallinity ensured the structural stability, and the small crystal size enabled a reduced Li^+^ ion diffusion distance.

Generally, in view of the anode materials, the incorporation of conductive materials such as MXene, graphene, and carbon nanotubes (CNTs) will greatly improve the electrochemical performances, owing to the facilitated charge transfer at the interface. Zhao et al. synthesized CoO/Co_2_Mo_3_O_8_@MXene hollow polyhedrons (Figure 15D,E) via chemical etching and evaluated their feasibility for LIBs as anode materials.^[^ ^152^ ^]^ The hollow polyhedron anode delivered discharge capacities of 1008.8, 875.8, 766.4, 658.2, 523.7, and 386.1 mAh g^–1^ at 0.1, 0.2, 0.5, 1, 2, and 5 mA g^–1^ (Figure 15F), respectively, implying an excellent rate performance. Furthermore, the capacity rapidly recovered to a high value of 936.2 mAh g^–1^ as the current density went back to 0.1 mA g^–1^. It was suggested that the unique combination of the CoO/Co_2_Mo_3_O_8_ and MXene nanosheets enabled a synergetic effect for enhancing the electrochemical performance of the anode. Particularly, the conductive MXene network promoted the interfacial charge transfer and accommodated the internal volume changes, while the CoO/Co_2_Mo_3_O_8_ prevented the aggregation of MXene nanosheets and provided the lithium storage capability. In addition, the interior voids in the hollow structure accommodated the structural stress during the repeated Li^+^ uptake and removal, rendering the electrode good cycling reliability. Remarkably, the CoO/Co_2_Mo_3_O_8_@MXene anode delivered 545 mAh g^–1^ at 2 A g^–1^ without any decay over 1200 cycles.

Multi‐shelled nanostructures are desired for lithium storage because of the enhanced packing density. For example, Sun et al. developed multi‐shelled transition‐metal oxide (TMO) hollow fibers as anode materials, and achieved high performances by optimizing the void spaces by controlling the shell numbers (Figure 15G,H).^[^ ^120^ ^]^ The NiO hollow microfibers with triple shells delivered 920.8 and 540.6 mAh g^–1^ at 0.1 and 5 A g^–1^, respectively (Figure 15I). The capacity recovered to 930.6 mAh g^–1^ as the current density dropped back to 0.1 A g^–1^. Comparably, inferior rate performances were obtained by the anodes based on single‐shelled (278.2 mAh g^–1^ at 5 A g^–1^), double‐shelled (385.9 mAh g^–1^ at 5 A g^–1^), and shell‐broken (211.4 mAh g^–1^ at 5 A g^–1^) NiO hollow microfibers. Apparently, the multi‐shelled structures are critical for enhancing the electrochemical performance of NiO hollow microfiber‐based anodes. In particular, the multiple shells with tunable voids can shorten the diffusion of Li^+^ and alleviate volume changes during cycling. Overall, the performances of the electrode materials with different hierarchical micro/nanostructures for LIBs are compared in Table 4.

**TABLE 4:** Electrochemical performances of different hierarchical micro/nanostructures prepared by self‐templating strategies for lithium‐ion batteries

Lithium‐ion batteries					
Hierarchical architectures	Electrode materials	Self‐templating synthetic mechanism	Highest reversible capacity	Cycling performance	Ref.
Hierarchically structured microframe with kinked surfaces	Fe_4_(Fe(CN)_6_)_3_	Chemical etching	730 mAh g^–1^ 30 mA g^–1^	430 mAh g^–1^, 5 C, 1000 cycles	[151]
Hollow polyhedron	CoO/Co_2_Mo_3_O_8_@MXene	Chemical etching	947.4 mAh g^–1^ 0.1 A g^–1^	545 mAh g^–1^, 2 A g^–1^, 1200 cycles	[152]
Multi‐shelled hollow fiber	NiO	Template contraction and transformation	698.1 mAh g^–1^ 1 A g^–1^	698.1 mAh g^–1^, 1 A g^–1^, 200 cycles	[120]
Hollow yolk–shell sphere	Sb@C	Galvanic replacement	634 mAh g^–1^ 0.1 A g^–1^	405 mAh g^–1^, 1 A g^–1^, 300 cycles	[107]
Yolk–shell microsphere	V_2_O_5_	Ostwald ripening	280 mAh g^–1^ 0.2 C	210 mAh g^–1^, 30 mA g^–1^, 30 cycles	[105]
Yolk–shell nanobox	FeP@C	Chemical etching	609 mAh g^–1^ 0.1 A g^–1^	476 mAh g^–1^, 0.5 A g^–1^, 400 cycles	[153]
Yolk–shell nanoprism	Ni‐Co Oxide	Template contraction and transformation	1025 mAh g^–1^ 0.2 A g^–1^	1028.5 mAh g^–1^, 0.2 A g^–1^, 30 cycles	[109]
Nanobubble hollow prism	CoS_2_	Template contraction and transformation	910 mAh g^–1^ 0.2 A g^–1^	737 mAh g^–1^, 1 A g^–1^, 200 cycles	[154]
Hollow octahedra	F‐CuO	Chemical etching	657 mAh g^–1^ 0.5 A g^–1^	624 mAh g^–1^, 1 A g^–1^, 300 cycles	[60]
Hollow sphere	SnO_2_	Kirkendall effect	638 mAh g^–1^ 10 A g^–1^	1043 mAh g^–1^, 3 A g^–1^, 500 cycles	[111]
Hollow nanotube	Sb@CTHNs	Galvanic replacement	700.6 mAh g^–1^ 50 mA g^–1^	607.2 mAh g^–1^, 100 mA g^–1^, 100 cycles	[155]
Hollow sphere	NiCo_2_O_4_	Template contraction and transformation	834 mAh g^–1^ 0.3 A g^–1^	706 mAh g^–1^, 0.2 A g^–1^, 100 cycles	[61]
Nanobox	H‐Co_3_O_4_@MCNBs	Chemical etching	1150 mAh g^–1^ 0.1 A g^–1^	1120 mAh g^–1^, 0.2 A g^–1^, 100 cycles	[114]

### 4.3 Other batteries

**FIGURE 16:**
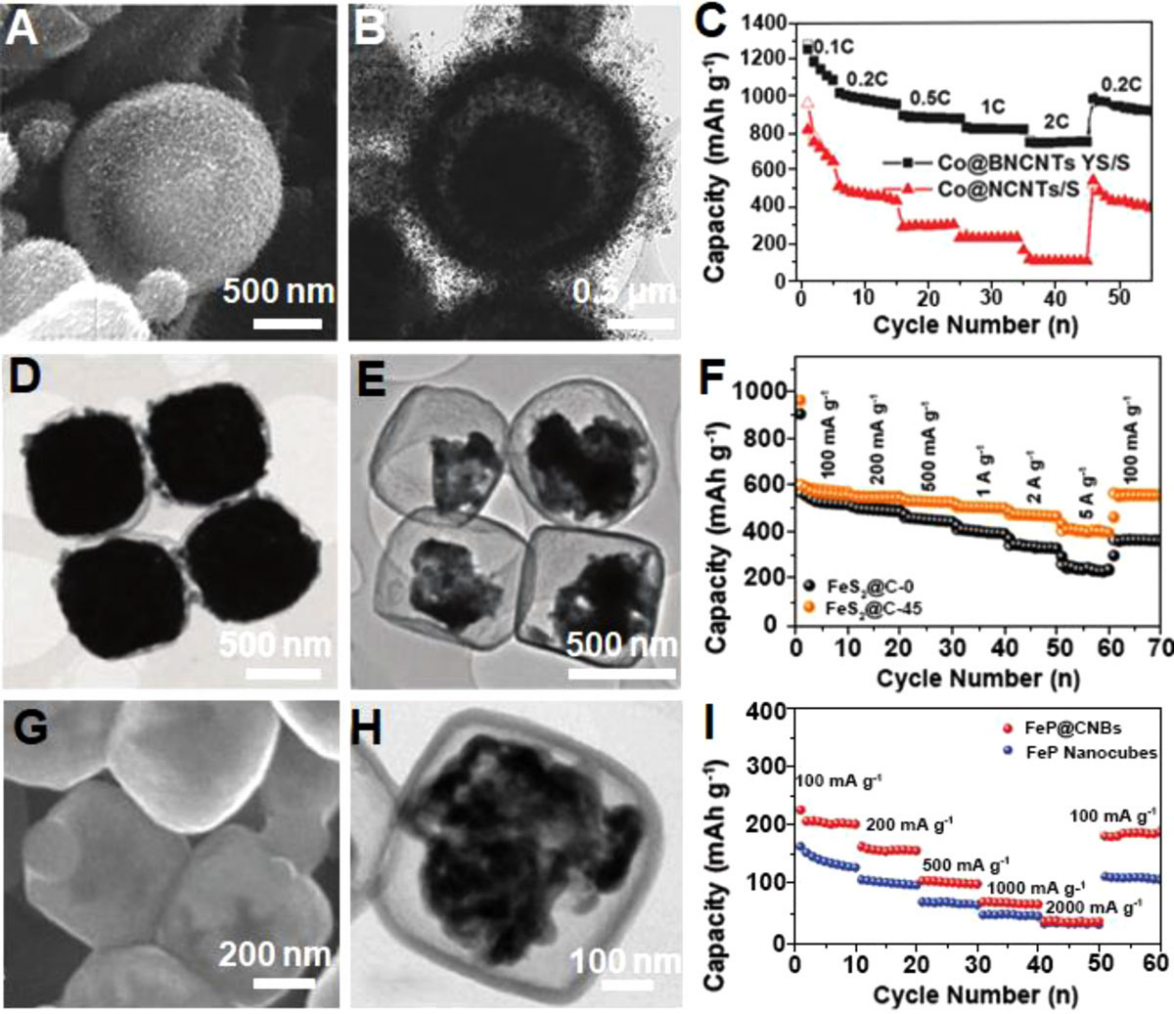
(A) SEM image, (B) TEM image, and (C) rate capabilities of the Co@BNCNTs yolk–shell microspheres. Reproduced with permission.^[^ ^177^ ^]^ Copyright 2017, Wiley‐VCH. (D,E) TEM images and (F) rate performances of FeS_2_@C‐0 and FeS_2_@C‐45 yolk–shell nanoboxes. Reproduced with permission.^[^ ^108^ ^]^ Copyright 2017, Royal Society of Chemistry. (G) SEM and (H) TEM image of the FeP@CNBs. (I) Rate performances of the FeP@C nanocubes and the FeP nanocubes as a function of current densities. Reproduced with permission.^[^ ^153^ ^]^ Copyright 2021, Wiley‐VCH

Recently, LSBs,^[^ ^156^, ^157^, ^158^, ^159^, ^160^ ^]^ SIBs,^[^ ^161^, ^162^, ^163^, ^164^, ^165^ ^]^ PIBs^[^ ^166^, ^167^, ^168^, ^169^, ^170^ ^]^ and others have demonstrated their potential as next‐generation EES devices. For example, the rich redox reactions between S and Li render LSBs with high energy density. Nevertheless, the low mass loading of sulfur, the high resistance of sulfur, and the shuttling of polysulfides severely hinder the practical applications of LSBs.^[^ ^171^, ^172^, ^173^ ^]^ Hence, it is crucial to introduce suitable hosts to boost the charge transfer and immobilize the polysulfides for enhancing the performances of LSBs.^[^ ^174^, ^175^, ^176^ ^]^ Park et al. employed hierarchical yolk–shell microspheres as cathode hosts for LSBs.^[^ ^177^ ^]^ The microspheres with yolk–shell structure were synthesized by thermal pyrolysis and the subsequent growth of N‐doped CNTs (Figure 16A,B). Co nanoparticles were encapsulated in N‐doped CNTs and the obtained Co@BNCNTs yolk–shell microspheres were then loaded with sulfur thus functioning as the cathode materials. The electrode respectively delivered stable capacities of 950, 874, 817, and 752 mAh g^–1^ at 0.2, 0.5, 1, and 2 C (Figure 16C). The unique structure promoted the electrolyte penetration, benefiting the sulfur storage and utilization, as well as increasing the electrode conductivity. Furthermore, the hierarchical yolk–shell microspheres rendered the electrode with 700.2 mAh g^–1^ retained after 400 cycles at 1 C. It was believed that the dissolution of polysulfides during cycling was effectively prevented due to the strong bonding between N‐doped C and S species.

In comparison with Li^+^ ions, Na^+^ and K^+^ ions have a larger radius, resulting in sluggish kinetics.^[^ ^11^, ^178^ ^]^ Structure nanoengineering is deemed as an effective method to confine the volume variation of electrode materials, shorten the electron/ion diffusion, and enhance the electrode conductivity for boosting the performance of electrodes. For instance, Liu et al. developed FeS_2_@C yolk–shell nanoboxes (Figure 16D,E) and applied as anode material for SIBs.^[^ ^108^ ^]^ The optimized FeS_2_@C anode that was chemically etched for 45 min (FeS_2_@C‐45) respectively delivered 560, 525, 502, and 403 mAh g^–1^ at 0.1, 0.5, 1, and 5 A g^–1^, with 556 mAh g^–1^ recovered when the current density dropped back to 0.1 A g^–1^ (Figure 16F). By contrast, FeS_2_@C without etching treatment (FeS_2_@C‐0) delivered inferior capacities, especially at elevated current densities. Besides, an irreversible capacity loss was observed as the current density returned to 0.1 A g^–1^ (Figure 16F). The large void space that can accommodate the volume expansion upon sodium intake alleviated the capacity fading issue; the porous yolks shortened the transports of electrons and Na^+^, thereby improving the reaction rate. Furthermore, because of the structural robustness and the protection by the carbon shells, the FeS_2_@C yolk–shell nanoboxes with etching time of 45 min delivered good cycling stability at 0.1 A g^–1^, with 511 mAh g^–1^ maintained after 100 cycles. FeP@carbon yolk–shell nanoboxes were employed as anode materials for PIBs as reported by Yang et al. (Figure 16G,H).^[^ ^153^ ^]^ The anode delivered stable capacities of 201, 156, 101, 65, and 37 mAh g^–1^ respectively at 0.1, 0.2, 0.5, 1, and 2 A g^–1^. As the current density returned to 0.1 A g^–1^, 200 mAh g^–1^ was recovered (Figure 16I). In addition, 205 mAh g^–1^ was achieved over 300 charge/discharge cycles at 0.1 A g^–1^, suggesting the good stability of FeP@carbon originated from their unique yolk–shell structures.

Metal‐air batteries, such as LABs and ZABs, have also drawn intense attention as novel EES devices for their ultrahigh theoretical energy densities.^[^ ^179^, ^180^, ^181^ ^]^ However, the practical performances of LABs and ZABs are strictly limited by cathodic electrodes which require high catalytic activities for oxygen reduction. Various nanostructures including pearl chain tube‐like composites of Ni_3_S_2_/N,S‐doped carbon, and yolk–shell Co_2_CrO_4_ nanospheres were designed as cathodic catalysts for LABs.^[^ ^182^, ^183^ ^]^ A high capacity of 16733.7 mAh g^–1^ at 0.4 A g^–1^ was stably achieved over 148 cycles. In the case of ZABs, Fe‐doped hollow‐structured mesoporous carbon (Fe/HOMC), Co/N‐C@NiCo_2_O_4_ hollow microspheres, and CoS_2_@MoS_2_@NiS_2_ double‐shell hollow polyhedrons were recently synthesized as cathodic catalysts.^[^ ^184^, ^185^, ^186^ ^]^ Comparably, the Fe/HOMC cathode exhibited a power density of 153 mW cm^–2^ at 300 mA cm^–2^ and outstanding cycling stability owing to enriched active sites and pathways for fast ion/gas diffusion. Overall, the electrochemical performances of the electrode materials with different hierarchical micro/nanostructures for SIBs, PIBs, LSBs, LABs, and ZABs are compared in Tables 5, 6, 7, 8, 9.

**TABLE 5:** Electrochemical performances of different hierarchical micro/nanostructures prepared by self‐templating strategies for sodium‐ion batteries

Sodium‐ion batteries					
Hierarchical architectures	Electrode materials	Self‐templating synthesis mechanism	Highest reversible capacity	Cycling performance	Ref.
Triple‐shelled nanobox	Sb@C@TiO_2_	Galvanic replacement	452 mAh g^–1^ 0.1 A g^–1^	193 mAh g^–1^, 1 A g^–1^, 4000 cycles	[187]
Hollow yolk–shell sphere	Sb@C	Galvanic replacement	548 mAh g^–1^ 0.1 A g^–1^	280 mAh g^–1^, 1 A g^–1^, 200 cycles	[107]
Yolk–shell nanobox	FeS_2_@C	Chemical etching	560 mAh g^–1^ 0.1 A g^–1^	330 mAh g^–1^, 2 A g^–1^, 800 cycles	[108]
Hollow nanotube	Sb@CTHNs	Galvanic replacement	640.9 mAh g^–1^ 50 mA g^–1^	607.2 mAh g^–1^, 100 mA g^–1^, 100 cycles	[155]
Hierarchically porous hollow nanosphere	Na_3_V_2_(PO4)_2_O_2_F	Ostwald ripening	124.7 mAh g^–1^ 1 C	100 mAh g^–1^, 1 C, 100 cycles	[188]
Nanotube	Sb	Galvanic replacement	546 mAh g^–1^ 0.1 A g^–1^	342 mAh g^–1^, 1 A g^–1^, 6000 cycles	[72]

**TABLE 6:** Electrochemical performances of different hierarchical micro/nanostructures prepared by self‐templating strategies for potassium‐ion batteries

Potassium‐ion batteries					
Hierarchical architectures	Electrode materials	Self‐templating synthesis mechanism	Highest reversible capacity	Cycling performance	Ref.
Yolk–shell nanobox	FeP@C	Chemical etching	205 mAh g^–1^ 0.1 A g^–1^	476 mAh g^–1^, 0.5 A g^–1^, 400 cycles	[153]
Microtube	Sb_2_Se_3_@C	Template contraction and transformation	312.8 mAh g^–1^ 0.1 A g^–1^	191.4 mAh g^–1^, 0.5 A g^–1^, 400 cycles	[88]
Core–shell nanorod	ZnSe@C	Template contraction and transformation	389.4 mAh g^–1^ 0.1 A g^–1^	204 mAh g^–1^, 2 A g^–1^, 100 cycles	[189]
Triple‐shelled hollow microspheres	MnCo_2_O_4_	Template contraction and transformation	259 mAh g^–1^ 0.1 A g^–1^	126 mAh g^–1^, 0.1 A g^–1^, 100 cycles	[190]
Hollow hierarchical porous olive‐like structure	HHPOC	Chemical etching	305.6 mAh g^–1^ 0.1 A g^–1^	N.A.	[191]

**TABLE 7:** Electrochemical performances of different hierarchical micro/nanostructures prepared by self‐templating strategies for lithium–sulfur batteries

Lithium‐sulfur batteries					
Hierarchical architectures	Electrode materials	Self‐templating synthesis mechanism	Highest reversible capacity	Cycling performance	Ref.
Yolk–shell structure	Co@BNCNTs	Template contraction and transformation	752 mAh g^–1^ 2 C	700.2 mAh g^–1^, 1 C, 400 cycles	[177]
Hollow nano‐spherical	NiSiO/NiS	Template contraction and transformation	466 mAh g^–1^ 2 C	640 mAh g^–1^, 0.5 C, 500 cycles	[192]
Semi‐hollow core–shell nanoparticles	S@Au@SiO_2_	Chemical etching	500 mAh g^–1^ 5 C	816 mAh g^–1^, 0.2 C, 100 cycles	[193]
Yolk–shell structure	S‐Pani	Template contraction and transformation	1101 mAh g^–1^ 0.2 C	765 mAh g^–1^, 0.2 C, 200 cycles	[194]
Hollow structure	Co_9_S_8_	Template contraction and transformation	897.7 mAh g^–1^ 0.2 C	540.5 mAh g^–1^, 0.5 C, 500 cycles	[195]

**TABLE 8:** Electrochemical performances of different hierarchical micro/nanostructures prepared by self‐templating strategies for lithium–air batteries

Lithium‐air batteries					
Hierarchical architectures	Electrode materials	Self‐templating synthesis mechanism	Highest reversible capacity	Cycling performance	Ref.
Core–shell structure	MnO_2_/Pd	Ostwald ripening	1220 mAh g^–1^ 0.1 mA cm^–2^	567.6 mAh g^–1^, 0.1 mA cm^–2^, 13 cycles	[196]
Dandelion‐like hollow structure	NiCo_2_O_4_	Template contraction and transformation	25,227 mAh g^–1^ 0.1 mA g^–1^	1000 mAh g^–1^, 0.4 mA g^–1^, 140 cycles	[197]
Nanotubes	Fe/Fe_3_C@garphitic carbon	Template contraction and transformation	6966 mAh g^–1^ 0.1 mA cm^–2^	500 mAh g^–1^, 0.1 mA cm^–2^, 37 cycles	[198]
Yolk–shell structure	Co_2_CrO_2_	Template contraction and transformation	N.A.	1000 mAh g^–1^, 0.2 mA g^–1^, 236 cycles	[183]
Pearl chain tube	Ni_3_S_2_@N, S‐PCT	Template contraction and transformation	16733.7 mAh g^–1^ 0.4 mA g^–1^	1000 mAh g^–1^, 0.45 mA g^–1^, 148 cycles	[182]

**TABLE 9:** Electrochemical performances of different hierarchical micro/nanostructures prepared by self‐templating strategies for zinc–air batteries

Zinc‐air batteries					
Hierarchical architectures	Electrode materials	Self‐templating synthesis mechanism	Specificcapacity	Peak power density	Ref.
Hollow architecture with mesoporous shells	HOMC	Template contraction and transformation	823 mAh g^–1^ 10 mA cm^–2^	153 mW cm^–2^	[184]
Hollow ball	Pt/CoFe_2_O_4_‐C	Template contraction and transformation	N.A.	N.A.	[199]
Hierarchical hollow	Co/N‐C@NiCo_2_O_4_	Chemical etching	713.9 mAh g^–1^ 10 mA cm^–2^	27.2–49.6 mW cm^–2^	[186]
Double‐shelled hollow polyhedron	CoS_2_@MoS_2_@NiS_2_	Chemical etching	N.A.	80.28 mW cm^–2^	[185]
Hierarchical porous structure	Fe‐NC SAC	Chemical etching	786 mAh g^–1^ 10 mA cm^–2^	180 mW cm^–2^	[200]

## 5 CONCLUSIONS AND OUTLOOK

The rapid development of novel fabrication methods to construct complex micro/nanostructures for efficient energy storage applications has been witnessed in the last two decades. In comparison with the traditional hard‐/soft‐templating approaches, the self‐templating strategy that directly converts the precursor templates into target architectures is more convenient and low cost for scale‐up production by eliminating cumbersome template removal processes. In addition, the well‐defined structures of the precursor templates allow for the regulation of the derivatives, enabling a uniform and reproducible synthesis of hierarchical micro/nanostructures with designable composition and morphology.

To better master the design of hierarchical micro/nanostructures through self‐templating routes, it is of importance to grasp the synthetic mechanisms thoroughly, and comprehensively evaluate the advantages/disadvantages of each method for the target architecture. Generally, Ostwald ripening is an effective strategy to construct hollow structures of large size (normally on microscale or sub‐microscale), it remains challenging to extend its availability down to nanometer level. The nanoscale Kirkendall effect has demonstrated its versatility to transform solid nanocrystals into hollow forms with core–shell, yolk–shell, single‐shelled hollow, multi‐shelled and nanoframe configurations. One distinctive feature of this method is its capability to synthesize highly crystalline hollow nanocrystals with a high yield, thus offering a reliable way to design structures with crystallinity‐dependent properties. Galvanic replacement reaction is powerful to prepare hollow metal crystals with tunable compositions; however, in most cases, the stability of the products needs to be further improved. Chemical etching is facile to fabricate heterogeneous hierarchical structures due to its selective reaction nature. Therefore, it is normally adopted for designing hollow structures with multiple components. The template contraction and transformation mechanism play an important role in producing multi‐shelled structures with adjustable element gradients. From the synthetic point of view, to construct the hierarchical micro/nanostructures with higher complexity in terms of geometry, composition, and interior architectures, a facile manner is to combine different self‐templating methods so as to take advantage of their synergetic effects. Meanwhile, developing few‐step self‐templating synthetic method can largely expand its versatility to novel precursor templating materials. Nevertheless, the research on such synthetic method requires more effort to advance the controllable preparation of diverse functional materials with desired hierarchical micro/nanostructures.

The increased complexity of the hierarchical micro/nanostructures has prompted the fine modulation of material properties, which in turn promotes their EES applications. Although many reports have demonstrated the enhanced electrochemical performances enabled by the complex micro/nanostructures, there still lacks a comprehensive understanding on how the designed architectures and the chemical compositions contribute to the charge storage performance of electrode materials. Furthermore, the multiple interfaces including electrode/electrolyte, core/shell, and the junctions between different components also are significantly important in guiding the efficient transport of ions and electrons. Therefore, continuous efforts are desired to build complex micro/nanostructures with complementary functionalities on demand according to the respective charge storage mechanisms of each type of EES devices and the properties of the adopted electrode materials by selecting an appropriate synthetic route. Last but not least, challenges remain in practically utilizing hierarchical micro/nanostructured materials in commercial devices. Hence, it is of necessity to exploit innovative techniques with the merits of cost‐effective and large‐scale manufacture which is not only suitable for conventional rigid devices but also for flexible ones.

## CONFLICT OF INTEREST

The authors declare no conflict of interest.
